# Crystal structures of two PCN pincer iridium complexes and one PCP pincer carbodi­phospho­rane iridium inter­mediate: substitution of one phosphine moiety of a carbodi­phospho­rane by an organic azide

**DOI:** 10.1107/S2056989018017644

**Published:** 2019-01-01

**Authors:** Gabriel Julian Partl, Felix Nussbaumer, Walter Schuh, Holger Kopacka, Klaus Wurst, Paul Peringer

**Affiliations:** a Institute of General, Inorganic and Theoretical Chemistry, University of Innsbruck, A-6020 Innsbruck, Austria

**Keywords:** crystal structure, carbodi­phospho­rane, iridium, organic azide, reductive elimination, PCP pincer, non-innocent behaviour, PCN pincer

## Abstract

The structure of [Ir{(4-Cl-C_6_H_4_N_3_)C(dppm)-κ^3^ *P*,*C*,*N*}(dppm-κ^2^ *P*,*P*′)]Cl·1.5CH_2_Cl_2_·0.5C_7_H_8_ (C_57_H_48_Cl_2_IrN_3_P_4_·1.5CH_2_Cl_2_·0.5C_7_H_8_) (**2**), dppm = bis­(di­phenyl­phosphino)methane {systematic name: [7-(4-chloro­phen­yl)-1,1,3,3-tetra­phenyl-5,6,7-tri­aza-κ*N* ^7^-1,3λ^4^-diphospha-κ*P* ^1^-hepta-4,6-dien-4-yl][methyl­ene­bis(di­phenyl­phosphine)-κ^2^ *P*,*P*′]iridium(I) chloride–di­chloro­methane–toluene (2/3/1)}, resulting from the reaction of [IrClH{C(dppm)_2_-κ^3^ *P*,*C*,*P*)(MeCN)]Cl (**1a**) with 1-azido-4-chloro­benzene, shows a monocationic five-coordinate Ir^I^ complex with a distorted trigonal–bipyramidal geometry. In **2**, the iridium centre is coordinated by the neutral triazeneyl­idene­phospho­rane (4-Cl-C_6_H_4_N_3_)C(dppm) acting as a PCN pincer ligand, and a chelating dppm unit. The structure of the coordination compound [IrCl(CN)H(C(dppm)_2_-κ^3^ *P*,*C*,*P*)]·CH_3_CN, (C_52_H_45_ClIrNP_4_·CH_3_CN) (**1b**) [systematic name: chlorido­cyanidohydrido(1,1,3,3,5,5,7,7-octa­phenyl-1,3λ^5^,5λ^4^,7-tetra­phospha-κ^2^ *P* ^1^,*P* ^7^-hept-3-en-4-yl)iridium(III) aceto­nitrile monosolvate], prepared from **1a** and KCN, reveals an octa­hedral Ir^III^ central atom with a meridional PCP pincer carbodi­phospho­rane (CDP) ligand; the chloride ligand is located *trans* to the central carbon of the CDP functionality while the hydrido and cyanido ligands are situated *trans* to each other. The chiral coordination compound [Ir(CN)((4-Cl-C_6_H_4_N_3_)CH(CH(P(Ph)_2_)_2_)-κ^3^ *P*,*C*,*N*)(dppm-κ^2^ *P*,*P*′)]·2CH_3_OH, (C_58_H_48_ClIrN_4_P_4_·2CH_3_OH) (**3**) (systematic name: {4-[3-(4-chloro­phen­yl)triazenido-κ*N* ^3^]-1,1,3,3-tetra­phenyl-1,3λ^5^-diphospha-κ*P* ^1^-but-2-en-4-yl}cyanido[methyl­enebis(di­phenyl­phosphine)-κ^2^ *P*,*P*′]iridium(III) methanol disolvate), formed via prolonged reaction of 1-azido-4-chloro­benzene with **1b**, features a six-coordinate Ir^III^ central atom. The iridium centre is coordinated by the dianionic *facial* PCN pincer ligand [(4-Cl-C_6_H_4_N_3_)CH(CH(P(Ph_2_)_2_)_2_)], a cyanido ligand *trans* to the central carbon of the PCN pincer ligand and a chelating dppm unit. Complex **2** exhibits a 2:1 positional disorder of the Cl^−^ anion. The CH_2_Cl_2_ and C_7_H_8_ solvent mol­ecules show occupational disorder, with the toluene mol­ecule exhibiting additional 1:1 positional disorder with some nearly overlying carbon atoms.

## Abstract 2

The syntheses and crystal structures of two PCN pincer iridium complexes, prepared from the reaction of their respective PCP pincer carbodi­phospho­rane iridium precursors with an organic azide, are reported. Crystal data for one of the precursors is also discussed.

## Chemical context   

Carbodi­phospho­ranes (CDPs), also termed double ylides, consist of two tertiary phosphines connected to a central divalent carbon(0) atom. The P—C bonds are best described as donor–acceptor inter­actions (Petz & Frenking, 2010 ▸). Most of the chemistry associated with CDPs concerns compounds with Lewis acids. Since the central CDP carbon possesses two lone electron pairs, it is therefore able to inter­act with either one or two Lewis acids (Chauvin & Canac, 2010 ▸; Petz & Frenking, 2010 ▸). Reactions involving the cleavage of the P—C bonds of the CDP functionality are less common in contrast to phospho­rus ylides (Petz & Frenking, 2010 ▸; Kolodiazhnyi, 1999 ▸). We hereby report the non-innocent reactivity (Poverenov & Milstein, 2013 ▸) of a PCP pincer ligand, whose central carbon is part of a CDP functionality, with an organic azide in the coordination sphere of iridium.

Treatment of the Ir^III^ PCP pincer CDP complex [Ir(Cl)(H)(C(dppm)_2_-κ^3^ *P*,*C*,*P*)(MeCN)]Cl (**1a**) (Schlapp-Hackl *et al.*, 2018 ▸) with 1-azido-4-chloro­benzene affords the Ir^I^ complex [Ir((4-Cl-C_6_H_4_N_3_)C(dppm)-κ^3^ *P*,*C*,*N*)(dppm-κ^2^ *P*,*P*′)]Cl (**2**). The reaction implies the substitution of one phosphine moiety of the PCP pincer ligand C(dppm)_2_ for the organic azide, thus producing the triazeneyl­idene­phospho­rane (4-Cl-C_6_H_4_N_3_)C(dppm), which acts as a PCN pincer ligand in **2**. The phosphine displaced from the CDP functionality ends up in the coordination sphere of iridium and becomes part of a four-membered dppm chelate ring (see scheme).
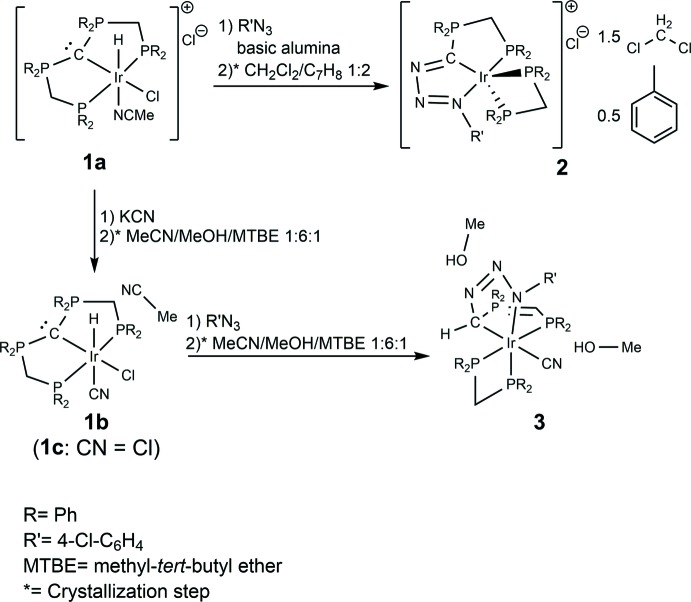


We believe that the reaction is initiated by an inter­action of the electrophilic organic azide with the central CDP carbon of the PCP ligand, which disposes of one lone electron pair (Petz & Frenking, 2010 ▸). In a related reaction, N-heterocyclic carbenes (NHCs) have been reported to form end-on adducts with organic azides to form triazenes (Khramov & Bielawski, 2005 ▸). The inter­action of the CDP with the organic azide results in the formation of a double bond between the central carbon and the terminal nitro­gen of the organic azide and is associated with the cleavage of one P—C bond of the CDP functionality, while the carbon–iridium bond remains intact. At this stage, a deeply coloured and presumably five-coord­in­ate Ir^I^ inter­mediate was detected by monitoring the reaction via ^31^P-NMR spectroscopy. This inter­mediate features the triazenyl­idene­phospho­rane ligand (4-Cl-C_6_H_4_N_3_)C(dppm) and a monodentate dppm unit. The absence of a hydrido ligand is attributed to an antecedent reductive elimination of hydro­chloric acid, which, according to NMR spectroscopic results, is absorbed by the CDP carbon of the starting complex **1a**. This carbon atom turns out to be the strongest base of the system, apparently more basic than the nitro­gen atoms and the central carbon of the PCN pincer ligand of **2**. Consequently, only 50% of the educt is converted to **2**. However, an almost qu­anti­tative and fast conversion into **2** was achieved upon addition of basic alumina. The formation of **2** is finalized via the dissociation of a chlorido ligand and the coordination of the displaced phosphine functionality to the Ir centre.

Treatment of **1a** with KCN affords [Ir(Cl)(CN)(H)((C(dppm)_2_)-κ^3^ *P*,*C*,*P*)] (**1b**), which reacts very slowly (over the course of weeks at room temperature) with 1-azido-4-chloro­benzene to the six-coordinate complex [Ir(CN)((4-Cl-C_6_H_4_N_3_)CH(CH(PPh_2_)_2_)-κ^3^ *P*,*C*,*N*)(dppm-κ^2^ *P*,*P*′)] (**3**). Related to the formation of **2**, the organic azide substitutes one phosphine of the CDP functionality.

At first sight, the resulting PCN pincer ligand of **3** looks like a tautomer of the PCN pincer ligand of **2**: while in the PCN pincer of **3**, one proton is attached to C1 and C2 respectively, the PCN pincer of **2** carries two protons at C2 and none at C1. In contrast to the neutral ligand of **2**, the PCN ligand in **3** carries a double negative charge, deduced as follows: first, in view of coordination number 6, **3** constitutes an Ir^III^ complex. Second, the coordination compound **3** carries no charge. Third, the cyanido ligand contributes a −1 charge, and the iridium central atom a +3 charge. Since the dppm ligand is neutral, the charge of the PCN pincer ligand can be calculated to be −2. We suspect that the pathway of the reaction is similar to the formation of **2**, except that the cyanido ligand permanently stays in the coordination sphere of iridium. The coordination of the displaced phosphine functionality to the Ir^I^ centre is thought to induce a two-electron transfer from iridium to the PCN ligand related to an oxidative addition reaction, and to be followed by the transfer of one proton from C2 to C1.

## Structural commentary   

**Figure 1:**
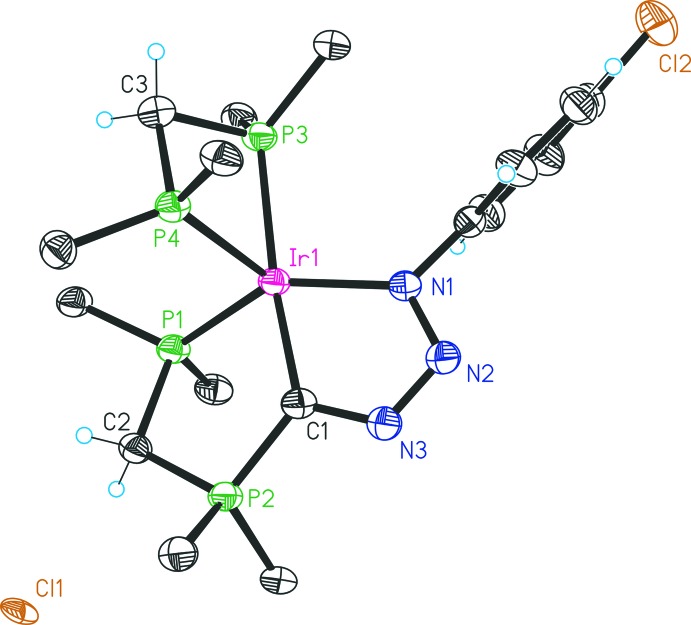
Structure of **2** with displacement ellipsoids drawn at the 30% probability level. For clarity, only the *ipso* carbon atoms of the phenyl groups are shown, and solvent mol­ecules have been omitted.

The structures of compounds **2**, **1b** and **3** are given in Figs. 1 ▸, 2 ▸ and 3 ▸, respectively. Selected bond lengths and angles for all three compounds are given in Table 1 ▸.

The structure of **2** (Fig. 1 ▸) features a cationic five-coordinate iridium(I) complex with a chloride counter-ion. The asymmetric unit additionally contains 1.5 mol­ecules of di­chloro­methane and half a mol­ecule of toluene. The iridium centre is coordinated by a PCN pincer ligand and a chelating dppm; its coordination sphere displays a distorted trigonal–bipyramidal geometry, in which the PCN pincer occupies one axial (C1) and two equatorial (P1 and N1) positions. The donor atoms of the chelating dppm are found in the remaining axial (P3) and equatorial (P4) positions. Major distortions are apparent from the angles P4—Ir1—P3 [70.69 (5)°] and N1—Ir1—P1 [140.4 (1)°], which reflect the ring strain of both the four-membered dppm chelate ring and the PCN pincer ligand. The angles around the ylidic carbon C1 cover a range of 115.9 (4) to 124.5 (3)°, with a sum total of 359°. The C1=N3 bond exhibits an increased length [1.346 (7) Å] relative to typical C=N double bonds (1.29 Å), resulting in a formal bond order (BO) of 1.7. With a bond length of 1.308 (6) Å, the N2—N3 bond’s BO is 1.7 as well; the N1—N2 distance amounts to 1.354 (6) Å (BO 1.5). In reported adducts of NHCs with organic azides, the C—N3 (numbering as in the free azides) bond lengths are similar to **2**, whereas N1—N2 separations are shorter (*ca* 1.27 Å) and the N2—N3 distances are longer (*ca* 1.35 Å) compared to the corresponding bond lengths in **2** (Khramov & Bielawski, 2005 ▸). These differences are presumably due to the coordination of N3 to the Ir centre. Organic azides themselves exhibit a short N2—N3 bond [e.g. 1.1322 (2) Å, BO 2.5, for 2,4,6-trichlorphenyl­azide], whereas the N1—N2 bond is distinctly longer [1.252 (2) Å, BO 1.9; Takayama *et al.*, 2010 ▸].

**Figure 2:**
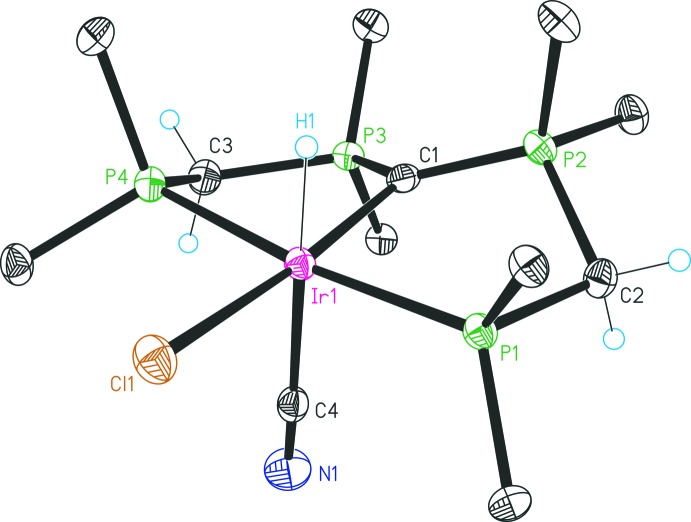
Structure of **1b** with displacement ellipsoids drawn at the 30% probability level. Only the *ipso* carbon atoms of the phenyl groups are shown for clarity, and solvent mol­ecules have been omitted.

The structure of **1b** (Fig. 2 ▸) displays an octa­hedral irid­ium(III) coordination compound with a *meridional* C(dppm)_2_ PCP pincer ligand and one chlorido ligand situated *trans* to the central CDP carbon atom. The remaining sites are occupied by the hydrido and cyanido ligands positioned *trans* to each other. The structure is closely related to that of [Ir(Cl)_2_(H)(C(dppm)_2_)-κ^3^ *P*,*C*,*P*)] (**1c**) (Partl *et al.*, 2018 ▸), which contains one chlorido ligand instead of the cyanido ligand *trans* to the hydrido ligand. The introduction of the cyanido ligand results in a markedly shorter Ir1—Cl1 bond *trans* to the CDP carbon [2.445 (1) Å compared to 2.5157 (14) Å for **1c**], whereas the Ir1—C1 separation becomes longer [2.128 (4)Å compared to 2.101 (5) Å]. Ir—P distances are marginally affected (Table 1 ▸).

**Figure 3:**
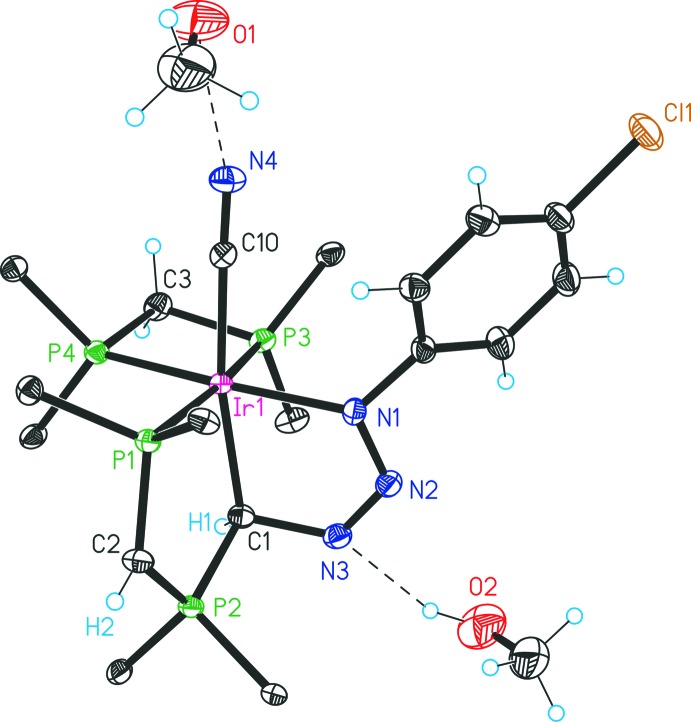
Structure of **3** with displacement ellipsoids drawn at the 30% probability level. Only the *ipso* carbon atoms of the phenyl groups are shown for clarity.

The structure of **3** (Fig. 3 ▸) shows a six-coordinate iridium(III) coordination compound with the PCN pincer ligand [(4-Cl-C_6_H_4_N_3_)CH(CH(PPh_2_)_2_)] in a *facial* mode, a bidentate dppm and a cyanido ligand *trans* to the central PCN carbon. Two mol­ecules of MeOH are connected to atoms N3 and N4 via hydrogen bonds. Distortions of the octa­hedral geometry are evident from the angles P3—Ir1—P4 and N1—Ir1—C1, amounting to 72.19 (2)° and 75.31 (9)°, respectively. The environment of the chiral carbon C1 is distorted tetra­hedral according to the angles N3—C1—Ir1 [109.8 (2)°], N3—C1—P2 [104.9 (2)°] and P2—C1—Ir1 [116.7 (1)°]. The deproton­ated dppm part of the PCN pincer ligand features delocal­ization over both P—C bonds [P1—C2 1.727 (3) and P2—C2 1.688 (3) Å, corresponding to a BO of *ca* 1.5 each). The C1—N3 bond is rather long [1.504 (3) Å, BO 0.8)] and appears to be in the range of protonated alkyl­amines, (Ishida, 2000 ▸) whereas the N2=N3 distance approximately corresponds to an N=N double bond [1.259 (3) Å, BO 1.9]. The N1—N2 separation is found to be in between a single and a double bond [1.347 (3) Å, BO 1.5].

## Supra­molecular features   

In the crystal of **2**, supra­molecular features appear to revolve around the chloride anion (Table 2 ▸): Cl1 interacts with the methyl­ene group of one dppm unit (C2—H2*B*⋯Cl1 = 2.62 Å) and to a proton of one di­chloro­methane mol­ecule (C11—H11*B*⋯Cl1^i^ = 2.49 Å). It must be mentioned, however, that due to the positional disorder of both the chloride anion and the di­chloro­methane solvate units, these ‘bond’ lengths are an estimation and may not necessarily reflect any actual inter­molecular inter­actions.

In the crystal of **1b**, the nitro­gen atom of the aceto­nitrile solvate interacts with the methyl­ene group of one dppm unit in a hydrogen-bond like manner (C3—H3*B*⋯N2 = 2.58 Å; Table 3 ▸). Inter­molecular halogen–hydrogen inter­actions are observed in two instances between phenyl protons and the chlorido ligand (C102—H102⋯Cl1 = 2.66 Å and C112—H112⋯Cl1^i^ = 2.77 Å; Table 3 ▸).

In the crystal of **3**, inter­molecular features are restricted to solvate coordination (Table 4 ▸): both methanol units are connected to the complex *via* hydrogen bonds, one to the triazenido group (O2—H2*A*⋯N3 = 2.16 Å) and the other to the cyanido ligand (O1—H1*A*⋯N4 = 2.02 Å).

## Synthesis and crystallization   

The syntheses of the title compounds are summarized in the general reaction scheme for the synthesis and crystallization of **2**, **1b** and **3**, starting from **1a** (**1c** is only mentioned for comparative purposes – see *Structural commentary*). All preparations were carried out under an inert atmosphere (N_2_) using standard Schlenk techniques. ^1^H, ^13^C and ^31^P NMR spectra were recorded on a Bruker DPX 300 NMR spectrometer (300 MHz) and were referenced against ^13^C/^1^H solvent peaks or an external 85% H_3_PO_4_ standard, respectively. The phospho­rus atoms in the NMR data are labelled in the same way as in the figures.

**Synthesis of complex 2: 1a** is formed upon stirring a mixture of [IrCl(cod)]_2_ (8.5 mg, 0.0125 mmol), [CH(dppm)_2_]Cl (20.5 mg, 0.025 mmol), (Reitsamer *et al.*, 2012 ▸), MeCN (0.1 ml) and MeOH (0.5 ml) for 25 min. After this, a solution of 1-azido-4-chloro­benzene (0.5 mol L^−1^ in methyl-*tert*-butyl ether, 0.1 ml, 0.050 mmol) and basic Al_2_O_3_ (30 mg) were subsequently added and the suspension was stirred for 5 min. The liquid part was separ­ated, and the volatiles evaporated *in vacuo*. The residue was then dissolved in CH_2_Cl_2_/toluene 1:2. Slow evaporation gave crystalline **2**.

^31^P{^1^H}-NMR (MeCN/MeOH 1:5): δ = 16.1 (P1, *ddd*; *J*_P1P2_ = 30.6, *J*_P1P3_ = 6.1, *J*_P1P4_ = 33.7 Hz); 15.6 (P2, *ddd*; *J*_P2P3_ = 44.4, *J*_P2P4_ = 13.8 Hz); −37.4 (P3, *ddd*; *J*_P3P4_ = 52.0 Hz); −20.9 (P4, *ddd*) ppm. ^13^C{^1^H}-NMR (MeCN/MeOH 1:5): δ = 169.5 (C1, *dddd*; *J*_C1P1_ = 2.5, *J*_C1P2_ = 60.0, *J*_C1P3_ = 85.0, *J*_C1P4_ = 8.3 Hz) ppm.

**Synthesis of coordination compounds 1b and 3:** A mixture of [IrCl(cod)]_2_ (8.5 mg, 0.0125 mmol), [CH(dppm)_2_]Cl (20.5 mg, 0.025 mmol), MeCN (0.1 ml) and MeOH (0.4 ml) was stirred for 25 min. Then, a solution of KCN (2 mg, 0.03 mmol) in MeOH (0.2 ml) was added and the mixture stirred for 1 min. Thereafter, a solution of 1-azido-4-chloro­benzene in MTBE (0.1 ml, mol L^−1^ in MTBE, 0.05 mmol) was added. Yellow crystals of **1b** formed within a few hours. Within 14 d, the orange crystals of **1b** disappeared, and colourless crystals of **3** had developed.

**1b** ^31^P{^1^H}-NMR (MeCN/MeOH 1:6): δ = −0.3 (P1/P4, *vt*, *N*_P1/P4P2/P3_ = 67.3 Hz); 28.2 (P2/P3, *vt*) ppm. ^13^C{^1^H}-NMR (MeCN/MeOH 1:6): δ = −36.5 (C1, *t*, *J*_C1P2/P3_ = 100.1 Hz) ppm. ^1^H-NMR (MeCN/MeOH 1:6): δ = −12.2 (H1, *s*, 1H) ppm.

**3** ^31^P{^1^H}-NMR (CHCl_3_/MeOH 1:1): δ = 0.3 (P1, *ddd*, *J*_P1P2_ = 153.0, *J*_P1P3_ = 376.3, *J*_P1P4_ = 18.4 Hz); 55.2 (P2, *dd*, *J*_P2P3_ = 49.0 Hz); −57.0 (P3, *ddd*, *J*_P3P4_ = 27.5 Hz); −67.1 (P4, *dd*) ppm. ^13^C-NMR (CHCl_3_/MeOH 1:1): δ = 72.2 (C1, *dd*, *J*_C1P2_ = 38.7, *J*_C1H1_ = 142.1 Hz) ppm.

## Refinement   

Crystal data, data collection and structure refinement details are summarized in Table 5 ▸. Complex **2** involves a 2:1 positional disorder of the anion Cl1:Cl1*A*. The solvent mol­ecules CH_2_Cl_2_ and C_7_H_8_ show occupational disorder, with the toluene mol­ecule exhibiting additional 1:1 positional disorder with some nearly overlying carbon atoms. We propose a correlation between CH_2_Cl_2_ and C_7_H_8_, because of short inter­molecular Cl⋯C contacts. Therefore, the two solvent mol­ecules Cl3/C10–Cl4 and Cl5/C11–Cl6 have an occupancy of 0.75 and the ‘two’ toluene mol­ecules, C12–C18 and C19–C25, an occupancy of 0.25. Several bond restraints were used to refine the toluene carbon atoms reasonably isotropically. The hydride hydrogen of **1b** was localized and refined isotropically without restraints. The hydrogen atoms at C1 and C2 of **3** were localized and refined with isotropic displacement parameters. All other H atoms were positioned geometrically (C—H = 0.94–0.98 Å) and refined as riding with *U*_iso_(H) = 1.2–1.5*U*_eq_(C).

## Supplementary Material

Crystal structure: contains datablock(s) global, 1b, 2, 3. DOI: 10.1107/S2056989018017644/su5464sup1.cif

Structure factors: contains datablock(s) 1b. DOI: 10.1107/S2056989018017644/su54641bsup2.hkl

Structure factors: contains datablock(s) 2. DOI: 10.1107/S2056989018017644/su54642sup3.hkl

Structure factors: contains datablock(s) 3. DOI: 10.1107/S2056989018017644/su54643sup4.hkl

CCDC references: 1880135, 1880134, 1880133

Additional supporting information:  crystallographic information; 3D view; checkCIF report

## Figures and Tables

**Table 1:** Selected bond lengths (Å) and angles (°) for **1b**, **2** and **3**

**1b**		**2**		**3**	
Ir1—C1	2.128 (4)	Ir1—C1	1.996 (5)	Ir1—C1	2.127 (3)
		Ir1—N1	1.999 (5)	Ir1—N1	2.109 (2)
Ir1—P1	2.302 (1)	Ir1—P1	2.2788 (14)	Ir1—P1	2.3595 (6)
Ir1—P4	2.277 (1)				
Ir1—Cl1	2.445 (1)	Ir1—P3	2.3748 (14)	Ir1—P3	2.3584 (7)
Ir1—C4	2.0815 (5)	Ir1—P4	2.2662 (14)	Ir1—P4	2.3304 (6)
Ir1—H1	1.51 (4)			Ir1—C10	2.026 (2)
P2—C1	1.689 (4)	P2—C1	1.776 (5)	P2—C1	1.843 (2)
C1—P3	1.689 (4)	C1—N3	1.346 (7)	C1—N3	1.505 (3)
		N1—N2	1.354 (6)	N1—N2	1.347 (3)
		N2—N3	1.308 (6)	N2—N3	1.259 (3)
					
C4—Ir1—P1	94.22 (12)	C1—Ir1—P1	85.35 (15)	C1—Ir1—P1	87.23 (7)
C1—Ir1—P1	89.10 (12)	N1—Ir1—P1	140.40 (13)	N1—Ir1—P1	96.69 (6)
P4—Ir1—P1	173.16 (4)	P4—Ir1—P1	95.59 (5)	P4—Ir1—P1	98.11 (2)
C4—Ir1—P4	89.08 (12)	C1—Ir1—P3	173.04 (16)	P3—Ir1—P1	169.15 (2)
C1—Ir1—P4	84.94 (12)	N1—Ir1—P3	100.49 (13)	C10—Ir1—P1	92.84 (7)
C4—Ir1—Cl1	94.42 (13)	P4—Ir1—P3	70.69 (5)	C10—Ir1—P3	92.00 (7)
C1—Ir1—Cl1	175.28 (12)	P1—Ir1—P3	101.62 (5)	N1—Ir1—P3	92.64 (6)
P4—Ir1—Cl1	92.17 (4)	C1—Ir1—P4	108.92 (16)	C1—Ir1—P3	89.80 (7)
P1—Ir1—Cl1	93.55 (4)	N1—Ir1—P4	122.71 (13)	P4—Ir1—P3	72.19 (2)
C4—Ir1—C1	89.27 (17)	C1—Ir1—N1	73.7 (2)	C10—Ir1—P4	89.88 (7)
C4—Ir1—H1	176.6 (13)			N1—Ir1—P4	164.45 (6)
				C1—Ir1—P4	100.63 (7)
				C10—Ir1—N1	94.15 (9)
				C10—Ir1—C1	169.38 (9)
				N1—Ir1—C1	75.31 (9)

**Table 2:** Hydrogen-bond geometry (Å, °) for **2** [Chem scheme1]

*D*—H⋯*A*	*D*—H	H⋯*A*	*D*⋯*A*	*D*—H⋯*A*
C2—H2*B*⋯Cl1	0.98	2.62	3.5215 (1)	153
C11—H11*B*⋯Cl1^i^	0.98	2.49	3.4594 (1)	170
C209—H209⋯N3^ii^	0.94	2.60	3.3722 (1)	140
C306—H306⋯Cl1*A* ^iii^	0.94	2.81	3.7037 (1)	159

Symmetry codes: (i) 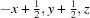; (ii) 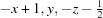; (iii) 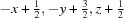.

**Table 3:** Hydrogen-bond geometry (Å, °) for **1b** [Chem scheme1]

*D*—H⋯*A*	*D*—H	H⋯*A*	*D*⋯*A*	*D*—H⋯*A*
C3—H3*B*⋯N2	0.98	2.58	3.4925 (1)	156
C102—H102⋯Cl1	0.94	2.66	3.5216 (1)	153
C112—H112⋯Cl1^i^	0.94	2.77	3.6470 (1)	155
C210—H210⋯N2^ii^	0.94	2.61	3.4554 (1)	150
C303—H303⋯N1^iii^	0.94	2.48	3.2064 (1)	134

Symmetry codes: (i) 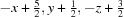; (ii) 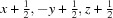; (iii) 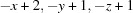.

**Table 4:** Hydrogen-bond geometry (Å, °) for **3** [Chem scheme1]

*D*—H⋯*A*	*D*—H	H⋯*A*	*D*⋯*A*	*D*—H⋯*A*
O1—H1*A*⋯N4	0.83	2.02	2.8181 (1)	162
O2—H2*A*⋯N3	0.83	2.16	2.9486 (1)	158

**Table 5:** Experimental details

	**1b**	**2**	**3**
Crystal data			
Chemical formula	[Ir(CN)ClH(C_51_H_44_P_4_)]·C_2_H_3_N	[Ir(C_25_H_22_P_2_)(C_32_H_26_ClN_3_P_2_)]Cl·0.5C_7_H_8_·1.5CH_2_Cl_2_	[Ir(CN)(C_23_H_22_P_2_)(C_34_H_26_ClN_3_P_2_)]·2CH_4_O
*M*_r_	1076.47	1335.42	1216.61
Crystal system, space group	Monoclinic, *P*2_1_/*n*	Orthorhombic, *P* *b* *c* *n*	Triclinic, *P* 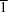
Temperature (K)	233	233	223
*a*, *b*, *c* (Å)	18.3264 (3), 13.6459 (2), 19.3010 (4)	28.1283 (3), 19.0989 (2), 23.6339 (2)	11.1683 (1), 12.7805 (2), 20.0591 (3)
α, β, γ (°)	90, 101.803 (1), 90	90, 90, 90	98.475 (1), 93.122 (1), 109.336 (1)
*V* (Å^3^)	4724.74 (14)	12696.6 (2)	2655.75 (6)
*Z*	4	8	2
Radiation type	Mo *K*α	Mo *K*α	Mo *K*α
μ (mm^−1^)	3.06	2.45	2.73
Crystal size (mm)	0.18 × 0.07 × 0.02	0.3 × 0.2 × 0.15	0.31 × 0.30 × 0.12

Data collection			
Diffractometer	Nonius KappaCCD	Nonius KappaCCD	Nonius KappaCCD
No. of measured, independent and observed [*I* > 2σ(*I*)] reflections	25915, 8317, 6345	73594, 11155, 9002	20072, 10396, 9895
*R*_int_	0.086	0.039	0.034
(sin θ/λ)_max_ (Å^−1^)	0.595	0.595	0.617

Refinement			
*R*[*F* ^2^ > 2σ(*F* ^2^)], *wR*(*F* ^2^), *S*	0.040, 0.069, 1.03	0.042, 0.126, 1.05	0.023, 0.056, 1.05
No. of reflections	8317	11155	10396
No. of parameters	564	724	661
No. of restraints	0	30	0
H-atom treatment	H atoms treated by a mixture of independent and constrained refinement	H-atom parameters constrained	H atoms treated by a mixture of independent and constrained refinement
Δρ_max_, Δρ_min_ (e Å^−3^)	0.56, −1.04	1.66, −0.73	0.77, −1.11

Computer programs: *COLLECT* (Nonius, 1998 ▸), *DENZO* and *SCALEPACK* (Otwinowski & Minor, 1997 ▸), *SHELXS97* (Sheldrick, 2008 ▸), *SHELXL2014* (Sheldrick, 2015 ▸), *Mercury* (Macrae *et al.*, 2008 ▸) and *publCIF* (Westrip, 2010 ▸).

## supplementary crystallographic information

### Chloridocyanidohydrido(1,1,3,3,5,5,7,7-octaphenyl-1,3λ5,5λ4,7-tetraphospha-κ2P1,P7-hept-3-en-4-yl)iridium(III) acetonitrile monosolvate (1b) Crystal data

**Table:**

[Ir(CN)ClH(C_51_H_44_P_4_)]·C_2_H_3_N	*F*(000) = 2160
*M**_r_* = 1076.47	*D*_x_ = 1.513 Mg m^−^^3^
Monoclinic, *P*2_1_/*n*	Mo *K*α radiation, λ = 0.71073 Å
*a* = 18.3264 (3) Å	Cell parameters from 52211 reflections
*b* = 13.6459 (2) Å	θ = 1.0–25.0°
*c* = 19.3010 (4) Å	µ = 3.06 mm^−^^1^
β = 101.803 (1)°	*T* = 233 K
*V* = 4724.74 (14) Å^3^	Prism, yellow
*Z* = 4	0.18 × 0.07 × 0.02 mm

### Chloridocyanidohydrido(1,1,3,3,5,5,7,7-octaphenyl-1,3λ5,5λ4,7-tetraphospha-κ2P1,P7-hept-3-en-4-yl)iridium(III) acetonitrile monosolvate (1b) Data collection

**Table:**

Nonius KappaCCD diffractometer	*R*_int_ = 0.086
phi– and ω–scans	θ_max_ = 25.0°, θ_min_ = 1.4°
25915 measured reflections	*h* = −21→20
8317 independent reflections	*k* = −16→16
6345 reflections with *I* > 2σ(*I*)	*l* = −22→22

### Chloridocyanidohydrido(1,1,3,3,5,5,7,7-octaphenyl-1,3λ5,5λ4,7-tetraphospha-κ2P1,P7-hept-3-en-4-yl)iridium(III) acetonitrile monosolvate (1b) Refinement

**Table:**

Refinement on *F*^2^	0 restraints
Least-squares matrix: full	Hydrogen site location: mixed
*R*[*F*^2^ > 2σ(*F*^2^)] = 0.040	H atoms treated by a mixture of independent and constrained refinement
*wR*(*F*^2^) = 0.069	*w* = 1/[σ^2^(*F*_o_^2^) + (0.021*P*)^2^ + 3.0711*P*] where *P* = (*F*_o_^2^ + 2*F*_c_^2^)/3
*S* = 1.03	(Δ/σ)_max_ = 0.003
8317 reflections	Δρ_max_ = 0.56 e Å^−^^3^
564 parameters	Δρ_min_ = −1.04 e Å^−^^3^

### Chloridocyanidohydrido(1,1,3,3,5,5,7,7-octaphenyl-1,3λ5,5λ4,7-tetraphospha-κ2P1,P7-hept-3-en-4-yl)iridium(III) acetonitrile monosolvate (1b) Special details

**Table:**

Experimental. All data sets were measured with several scans to increase the number of redundant reflections. In our experience this method of averaging redundant reflections replaces in a good approximation semi-empirical absorptions methods (absorption correction programs like SORTAV lead to no better data sets).
Geometry. All esds (except the esd in the dihedral angle between two l.s. planes) are estimated using the full covariance matrix. The cell esds are taken into account individually in the estimation of esds in distances, angles and torsion angles; correlations between esds in cell parameters are only used when they are defined by crystal symmetry. An approximate (isotropic) treatment of cell esds is used for estimating esds involving l.s. planes.
Refinement. Small crystal with low diffraction at higher 2 theta angles. Hydrogen at Ir were localized and refined isotropically without restraints.

### Chloridocyanidohydrido(1,1,3,3,5,5,7,7-octaphenyl-1,3λ5,5λ4,7-tetraphospha-κ2P1,P7-hept-3-en-4-yl)iridium(III) acetonitrile monosolvate (1b) Fractional atomic coordinates and isotropic or equivalent isotropic displacement parameters (Å^2^)

**Table:**

	*x*	*y*	*z*	*U*_iso_*/*U*_eq_	
Ir1	1.08853 (2)	0.20725 (2)	0.71435 (2)	0.02191 (7)	
H1	1.1054 (19)	0.172 (3)	0.790 (2)	0.020 (11)*	
P1	1.20178 (6)	0.28650 (9)	0.73888 (6)	0.0239 (3)	
P2	1.10074 (7)	0.40362 (9)	0.80500 (6)	0.0269 (3)	
P3	0.94640 (6)	0.35370 (9)	0.72144 (6)	0.0251 (3)	
P4	0.97290 (6)	0.13964 (9)	0.70074 (6)	0.0260 (3)	
Cl1	1.14187 (7)	0.05475 (9)	0.68044 (7)	0.0383 (3)	
C4	1.0599 (2)	0.2604 (3)	0.6113 (3)	0.0278 (11)	
N1	1.0392 (2)	0.2906 (3)	0.5559 (2)	0.0486 (12)	
C1	1.0385 (2)	0.3332 (3)	0.7501 (2)	0.0250 (11)	
C2	1.1831 (2)	0.4124 (3)	0.7662 (3)	0.0279 (11)	
H2A	1.2256	0.4375	0.8009	0.034*	
H2B	1.1738	0.4565	0.7252	0.034*	
C3	0.9069 (2)	0.2410 (3)	0.6795 (3)	0.0314 (12)	
H3A	0.8941	0.2502	0.6281	0.038*	
H3B	0.8610	0.2252	0.6958	0.038*	
C101	1.2526 (2)	0.3058 (3)	0.6676 (2)	0.0268 (11)	
C102	1.2477 (3)	0.2350 (4)	0.6151 (3)	0.0390 (13)	
H102	1.2189	0.1783	0.6167	0.047*	
C103	1.2852 (3)	0.2482 (4)	0.5605 (3)	0.0518 (16)	
H103	1.2825	0.1996	0.5256	0.062*	
C104	1.3261 (3)	0.3312 (4)	0.5567 (3)	0.0473 (15)	
H104	1.3509	0.3397	0.5191	0.057*	
C105	1.3309 (3)	0.4013 (4)	0.6076 (3)	0.0468 (15)	
H105	1.3589	0.4583	0.6046	0.056*	
C106	1.2951 (3)	0.3896 (4)	0.6638 (3)	0.0386 (13)	
H106	1.2994	0.4380	0.6991	0.046*	
C107	1.2722 (2)	0.2390 (3)	0.8118 (2)	0.0260 (11)	
C108	1.2622 (3)	0.1486 (4)	0.8407 (3)	0.0377 (13)	
H108	1.2199	0.1111	0.8210	0.045*	
C109	1.3127 (3)	0.1120 (4)	0.8979 (3)	0.0500 (15)	
H109	1.3051	0.0499	0.9164	0.060*	
C110	1.3737 (3)	0.1661 (4)	0.9276 (3)	0.0468 (15)	
H110	1.4071	0.1427	0.9678	0.056*	
C111	1.3864 (3)	0.2544 (4)	0.8988 (3)	0.0451 (14)	
H111	1.4295	0.2902	0.9183	0.054*	
C112	1.3361 (2)	0.2916 (4)	0.8411 (3)	0.0380 (12)	
H112	1.3452	0.3525	0.8217	0.046*	
C201	1.0711 (3)	0.5264 (4)	0.8215 (3)	0.0333 (12)	
C202	1.0762 (3)	0.6032 (4)	0.7762 (3)	0.0463 (14)	
H202	1.0988	0.5933	0.7372	0.056*	
C203	1.0482 (3)	0.6950 (4)	0.7877 (4)	0.0652 (18)	
H203	1.0515	0.7468	0.7564	0.078*	
C204	1.0159 (3)	0.7098 (5)	0.8444 (4)	0.0659 (19)	
H204	0.9963	0.7718	0.8515	0.079*	
C205	1.0116 (4)	0.6357 (5)	0.8910 (4)	0.071 (2)	
H205	0.9900	0.6469	0.9305	0.085*	
C206	1.0394 (3)	0.5437 (4)	0.8798 (3)	0.0479 (15)	
H206	1.0367	0.4927	0.9119	0.058*	
C207	1.1346 (3)	0.3515 (4)	0.8923 (3)	0.0338 (12)	
C208	1.0916 (3)	0.2794 (4)	0.9159 (3)	0.0468 (14)	
H208	1.0472	0.2581	0.8864	0.056*	
C209	1.1141 (4)	0.2391 (5)	0.9828 (3)	0.0690 (19)	
H209	1.0840	0.1918	0.9987	0.083*	
C210	1.1788 (4)	0.2670 (5)	1.0253 (3)	0.073 (2)	
H210	1.1942	0.2378	1.0700	0.088*	
C211	1.2217 (4)	0.3377 (6)	1.0034 (3)	0.0672 (19)	
H211	1.2661	0.3577	1.0336	0.081*	
C212	1.2001 (3)	0.3807 (4)	0.9364 (3)	0.0499 (15)	
H212	1.2300	0.4291	0.9215	0.060*	
C301	0.9145 (2)	0.4436 (3)	0.6526 (2)	0.0277 (11)	
C302	0.9642 (3)	0.4980 (4)	0.6233 (3)	0.0395 (13)	
H302	1.0157	0.4913	0.6408	0.047*	
C303	0.9379 (3)	0.5629 (4)	0.5678 (3)	0.0450 (14)	
H303	0.9720	0.6004	0.5486	0.054*	
C304	0.8632 (3)	0.5723 (4)	0.5411 (3)	0.0451 (14)	
H304	0.8458	0.6172	0.5045	0.054*	
C305	0.8135 (3)	0.5153 (4)	0.5686 (3)	0.0518 (16)	
H305	0.7621	0.5205	0.5498	0.062*	
C306	0.8386 (3)	0.4512 (4)	0.6230 (3)	0.0406 (14)	
H306	0.8044	0.4119	0.6406	0.049*	
C307	0.8966 (2)	0.3862 (4)	0.7904 (3)	0.0309 (12)	
C308	0.8912 (3)	0.3181 (4)	0.8417 (3)	0.0546 (17)	
H308	0.9099	0.2545	0.8382	0.066*	
C309	0.8585 (4)	0.3418 (5)	0.8983 (4)	0.081 (2)	
H309	0.8551	0.2943	0.9328	0.097*	
C310	0.8316 (4)	0.4334 (5)	0.9041 (4)	0.078 (2)	
H310	0.8102	0.4495	0.9429	0.094*	
C311	0.8354 (3)	0.5022 (5)	0.8537 (3)	0.0642 (18)	
H311	0.8160	0.5653	0.8574	0.077*	
C312	0.8682 (3)	0.4788 (4)	0.7965 (3)	0.0463 (14)	
H312	0.8710	0.5264	0.7620	0.056*	
C401	0.9435 (3)	0.0522 (4)	0.6292 (3)	0.0346 (13)	
C402	0.9211 (3)	0.0818 (4)	0.5597 (3)	0.0501 (15)	
H402	0.9182	0.1492	0.5494	0.060*	
C403	0.9029 (3)	0.0156 (5)	0.5046 (4)	0.0666 (19)	
H403	0.8876	0.0381	0.4579	0.080*	
C404	0.9073 (3)	−0.0821 (5)	0.5184 (4)	0.0650 (19)	
H404	0.8935	−0.1273	0.4813	0.078*	
C405	0.9314 (4)	−0.1145 (5)	0.5855 (4)	0.068 (2)	
H405	0.9358	−0.1821	0.5945	0.082*	
C406	0.9502 (3)	−0.0471 (4)	0.6425 (3)	0.0548 (16)	
H406	0.9671	−0.0700	0.6889	0.066*	
C407	0.9433 (3)	0.0784 (3)	0.7744 (3)	0.0332 (12)	
C408	0.9928 (3)	0.0573 (4)	0.8363 (3)	0.0524 (16)	
H408	1.0433	0.0738	0.8407	0.063*	
C409	0.9695 (4)	0.0124 (5)	0.8918 (3)	0.077 (2)	
H409	1.0043	−0.0032	0.9333	0.092*	
C410	0.8955 (4)	−0.0098 (5)	0.8868 (4)	0.083 (2)	
H410	0.8793	−0.0385	0.9253	0.099*	
C411	0.8463 (4)	0.0098 (5)	0.8266 (5)	0.084 (2)	
H411	0.7958	−0.0060	0.8232	0.101*	
C412	0.8689 (3)	0.0531 (4)	0.7694 (3)	0.0580 (17)	
H412	0.8340	0.0652	0.7273	0.070*	
N2	0.7247 (3)	0.2498 (6)	0.7077 (4)	0.103 (2)	
C5	0.6995 (4)	0.2484 (5)	0.7548 (5)	0.077 (2)	
C6	0.6679 (6)	0.2427 (8)	0.8163 (5)	0.158 (5)	
H6A	0.6629	0.1745	0.8287	0.237*	
H6B	0.6191	0.2735	0.8068	0.237*	
H6C	0.7000	0.2761	0.8553	0.237*	

### Chloridocyanidohydrido(1,1,3,3,5,5,7,7-octaphenyl-1,3λ5,5λ4,7-tetraphospha-κ2P1,P7-hept-3-en-4-yl)iridium(III) acetonitrile monosolvate (1b) Atomic displacement parameters (Å^2^)

**Table:**

	*U*^11^	*U*^22^	*U*^33^	*U*^12^	*U*^13^	*U*^23^
Ir1	0.02357 (11)	0.02054 (11)	0.02067 (10)	0.00045 (9)	0.00230 (7)	−0.00009 (9)
P1	0.0248 (6)	0.0235 (7)	0.0224 (6)	0.0009 (5)	0.0023 (5)	−0.0005 (6)
P2	0.0297 (7)	0.0253 (7)	0.0254 (7)	0.0017 (5)	0.0049 (5)	−0.0042 (6)
P3	0.0262 (7)	0.0263 (7)	0.0235 (7)	0.0017 (5)	0.0062 (5)	0.0006 (6)
P4	0.0261 (7)	0.0241 (7)	0.0268 (7)	−0.0007 (5)	0.0028 (5)	0.0003 (6)
Cl1	0.0443 (8)	0.0313 (7)	0.0376 (8)	0.0043 (6)	0.0040 (6)	−0.0016 (6)
C4	0.027 (3)	0.023 (3)	0.034 (3)	−0.001 (2)	0.008 (2)	−0.002 (2)
N1	0.063 (3)	0.050 (3)	0.031 (3)	0.001 (2)	0.004 (2)	0.013 (3)
C1	0.024 (3)	0.030 (3)	0.022 (3)	−0.002 (2)	0.007 (2)	0.002 (2)
C2	0.027 (3)	0.023 (3)	0.034 (3)	0.000 (2)	0.006 (2)	0.000 (2)
C3	0.032 (3)	0.030 (3)	0.032 (3)	−0.002 (2)	0.004 (2)	0.001 (2)
C101	0.025 (3)	0.029 (3)	0.027 (3)	0.004 (2)	0.007 (2)	0.008 (2)
C102	0.043 (3)	0.035 (3)	0.041 (3)	−0.008 (2)	0.014 (3)	−0.013 (3)
C103	0.068 (4)	0.056 (4)	0.039 (3)	−0.008 (3)	0.028 (3)	−0.018 (3)
C104	0.046 (4)	0.061 (4)	0.041 (4)	0.002 (3)	0.023 (3)	0.005 (3)
C105	0.048 (4)	0.044 (4)	0.056 (4)	−0.007 (3)	0.027 (3)	0.007 (3)
C106	0.039 (3)	0.032 (3)	0.046 (3)	−0.002 (2)	0.011 (3)	−0.005 (3)
C107	0.029 (3)	0.022 (3)	0.026 (3)	0.004 (2)	0.003 (2)	−0.001 (2)
C108	0.031 (3)	0.038 (3)	0.039 (3)	−0.004 (2)	−0.005 (2)	0.006 (3)
C109	0.049 (4)	0.048 (4)	0.045 (4)	−0.002 (3)	−0.010 (3)	0.017 (3)
C110	0.034 (3)	0.063 (4)	0.037 (3)	0.008 (3)	−0.008 (3)	0.005 (3)
C111	0.035 (3)	0.050 (4)	0.044 (3)	−0.001 (3)	−0.006 (3)	−0.007 (3)
C112	0.029 (3)	0.033 (3)	0.049 (3)	−0.004 (2)	0.001 (2)	−0.003 (3)
C201	0.031 (3)	0.030 (3)	0.038 (3)	0.001 (2)	0.005 (2)	−0.007 (3)
C202	0.045 (3)	0.037 (4)	0.057 (4)	0.005 (3)	0.012 (3)	−0.007 (3)
C203	0.073 (4)	0.035 (4)	0.087 (5)	0.011 (3)	0.017 (4)	0.000 (4)
C204	0.056 (4)	0.040 (4)	0.101 (6)	0.011 (3)	0.012 (4)	−0.028 (4)
C205	0.077 (5)	0.060 (5)	0.083 (5)	0.012 (4)	0.033 (4)	−0.033 (4)
C206	0.055 (4)	0.039 (3)	0.052 (4)	0.005 (3)	0.018 (3)	−0.013 (3)
C207	0.035 (3)	0.037 (3)	0.028 (3)	0.007 (2)	0.001 (2)	−0.007 (3)
C208	0.058 (4)	0.048 (4)	0.032 (3)	0.003 (3)	0.005 (3)	−0.002 (3)
C209	0.101 (6)	0.068 (5)	0.035 (4)	0.012 (4)	0.009 (4)	0.019 (3)
C210	0.099 (6)	0.088 (6)	0.029 (4)	0.032 (5)	0.003 (4)	0.008 (4)
C211	0.064 (4)	0.097 (6)	0.031 (4)	0.015 (4)	−0.012 (3)	−0.009 (4)
C212	0.049 (4)	0.068 (4)	0.031 (3)	0.000 (3)	0.004 (3)	−0.008 (3)
C301	0.027 (3)	0.027 (3)	0.028 (3)	0.003 (2)	0.005 (2)	0.002 (2)
C302	0.032 (3)	0.043 (3)	0.043 (3)	−0.002 (2)	0.005 (2)	0.012 (3)
C303	0.053 (4)	0.042 (3)	0.039 (3)	−0.006 (3)	0.009 (3)	0.019 (3)
C304	0.055 (4)	0.042 (3)	0.036 (3)	0.009 (3)	0.005 (3)	0.017 (3)
C305	0.038 (3)	0.066 (4)	0.049 (4)	0.014 (3)	0.003 (3)	0.017 (3)
C306	0.035 (3)	0.045 (3)	0.043 (3)	0.008 (2)	0.008 (3)	0.015 (3)
C307	0.028 (3)	0.034 (3)	0.031 (3)	0.005 (2)	0.005 (2)	−0.001 (3)
C308	0.070 (4)	0.050 (4)	0.055 (4)	0.018 (3)	0.037 (3)	0.011 (3)
C309	0.136 (7)	0.060 (5)	0.065 (5)	0.022 (4)	0.064 (5)	0.022 (4)
C310	0.118 (6)	0.076 (5)	0.056 (5)	0.015 (5)	0.057 (4)	0.004 (4)
C311	0.081 (5)	0.058 (4)	0.062 (4)	0.022 (3)	0.034 (4)	−0.011 (4)
C312	0.056 (4)	0.045 (4)	0.040 (3)	0.013 (3)	0.016 (3)	0.007 (3)
C401	0.025 (3)	0.034 (3)	0.045 (3)	−0.007 (2)	0.008 (2)	−0.012 (3)
C402	0.056 (4)	0.055 (4)	0.036 (3)	−0.013 (3)	0.002 (3)	−0.017 (3)
C403	0.059 (4)	0.085 (6)	0.054 (4)	−0.014 (4)	0.009 (3)	−0.032 (4)
C404	0.064 (4)	0.063 (5)	0.065 (5)	−0.012 (4)	0.007 (4)	−0.035 (4)
C405	0.070 (4)	0.033 (4)	0.104 (6)	−0.009 (3)	0.023 (4)	−0.026 (4)
C406	0.057 (4)	0.039 (4)	0.067 (4)	−0.001 (3)	0.009 (3)	−0.002 (3)
C407	0.035 (3)	0.029 (3)	0.037 (3)	−0.002 (2)	0.009 (2)	0.007 (3)
C408	0.044 (4)	0.063 (4)	0.052 (4)	0.001 (3)	0.015 (3)	0.024 (3)
C409	0.069 (5)	0.109 (6)	0.055 (4)	0.004 (4)	0.019 (3)	0.047 (4)
C410	0.077 (5)	0.094 (6)	0.090 (6)	0.002 (4)	0.048 (5)	0.049 (5)
C411	0.057 (5)	0.092 (6)	0.117 (7)	−0.007 (4)	0.048 (5)	0.042 (5)
C412	0.039 (4)	0.063 (4)	0.073 (5)	−0.007 (3)	0.013 (3)	0.024 (4)
N2	0.065 (4)	0.171 (7)	0.072 (5)	−0.012 (4)	0.011 (4)	−0.009 (5)
C5	0.088 (6)	0.066 (5)	0.082 (6)	0.003 (4)	0.029 (5)	−0.008 (5)
C6	0.197 (11)	0.182 (11)	0.126 (9)	−0.015 (9)	0.104 (9)	−0.010 (8)

### Chloridocyanidohydrido(1,1,3,3,5,5,7,7-octaphenyl-1,3λ5,5λ4,7-tetraphospha-κ2P1,P7-hept-3-en-4-yl)iridium(III) acetonitrile monosolvate (1b) Geometric parameters (Å, º)

**Table:**

Ir1—P1	2.3020 (12)	C207—C208	1.395 (7)
Ir1—P4	2.2766 (12)	C208—C209	1.385 (7)
Ir1—C1	2.128 (4)	C208—H208	0.9400
Ir1—Cl1	2.4446 (12)	C209—C210	1.352 (9)
Ir1—C4	2.081 (5)	C209—H209	0.9400
Ir1—H1	1.51 (4)	C210—C211	1.365 (9)
P1—C107	1.823 (5)	C210—H210	0.9400
P1—C101	1.831 (4)	C211—C212	1.402 (8)
P1—C2	1.850 (4)	C211—H211	0.9400
P2—C1	1.690 (5)	C212—H212	0.9400
P2—C201	1.810 (5)	C301—C302	1.382 (6)
P2—C207	1.817 (5)	C301—C306	1.394 (6)
P2—C2	1.821 (4)	C302—C303	1.398 (7)
P3—C1	1.688 (4)	C302—H302	0.9400
P3—C301	1.816 (5)	C303—C304	1.366 (7)
P3—C307	1.817 (5)	C303—H303	0.9400
P3—C3	1.818 (5)	C304—C305	1.385 (7)
P4—C401	1.822 (5)	C304—H304	0.9400
P4—C407	1.826 (5)	C305—C306	1.372 (7)
P4—C3	1.828 (5)	C305—H305	0.9400
C4—N1	1.136 (6)	C306—H306	0.9400
C2—H2A	0.9800	C307—C308	1.375 (7)
C2—H2B	0.9800	C307—C312	1.380 (7)
C3—H3A	0.9800	C308—C309	1.388 (7)
C3—H3B	0.9800	C308—H308	0.9400
C101—C102	1.391 (6)	C309—C310	1.357 (9)
C101—C106	1.393 (6)	C309—H309	0.9400
C102—C103	1.382 (7)	C310—C311	1.365 (8)
C102—H102	0.9400	C310—H310	0.9400
C103—C104	1.368 (7)	C311—C312	1.397 (7)
C103—H103	0.9400	C311—H311	0.9400
C104—C105	1.360 (7)	C312—H312	0.9400
C104—H104	0.9400	C401—C406	1.379 (7)
C105—C106	1.389 (7)	C401—C402	1.381 (7)
C105—H105	0.9400	C402—C403	1.383 (7)
C106—H106	0.9400	C402—H402	0.9400
C107—C108	1.382 (6)	C403—C404	1.358 (9)
C107—C112	1.391 (6)	C403—H403	0.9400
C108—C109	1.381 (7)	C404—C405	1.355 (9)
C108—H108	0.9400	C404—H404	0.9400
C109—C110	1.365 (7)	C405—C406	1.420 (8)
C109—H109	0.9400	C405—H405	0.9400
C110—C111	1.366 (7)	C406—H406	0.9400
C110—H110	0.9400	C407—C408	1.374 (7)
C111—C112	1.390 (7)	C407—C412	1.389 (7)
C111—H111	0.9400	C408—C409	1.376 (7)
C112—H112	0.9400	C408—H408	0.9400
C201—C202	1.381 (7)	C409—C410	1.374 (8)
C201—C206	1.388 (7)	C409—H409	0.9400
C202—C203	1.388 (7)	C410—C411	1.344 (9)
C202—H202	0.9400	C410—H410	0.9400
C203—C204	1.361 (9)	C411—C412	1.388 (8)
C203—H203	0.9400	C411—H411	0.9400
C204—C205	1.367 (9)	C412—H412	0.9400
C204—H204	0.9400	N2—C5	1.101 (8)
C205—C206	1.388 (8)	C5—C6	1.427 (10)
C205—H205	0.9400	C6—H6A	0.9700
C206—H206	0.9400	C6—H6B	0.9700
C207—C212	1.380 (7)	C6—H6C	0.9700
			
C4—Ir1—C1	89.27 (17)	C204—C205—C206	119.6 (6)
C4—Ir1—P4	89.08 (12)	C204—C205—H205	120.2
C1—Ir1—P4	84.94 (12)	C206—C205—H205	120.2
C4—Ir1—P1	94.22 (12)	C201—C206—C205	120.5 (6)
C1—Ir1—P1	89.10 (12)	C201—C206—H206	119.7
P4—Ir1—P1	173.16 (4)	C205—C206—H206	119.7
C4—Ir1—Cl1	94.42 (13)	C212—C207—C208	118.7 (5)
C1—Ir1—Cl1	175.28 (12)	C212—C207—P2	123.2 (4)
P4—Ir1—Cl1	92.17 (4)	C208—C207—P2	118.1 (4)
P1—Ir1—Cl1	93.55 (4)	C209—C208—C207	120.3 (6)
C4—Ir1—H1	176.6 (13)	C209—C208—H208	119.8
C1—Ir1—H1	87.8 (14)	C207—C208—H208	119.8
P4—Ir1—H1	89.0 (13)	C210—C209—C208	120.7 (7)
P1—Ir1—H1	87.4 (13)	C210—C209—H209	119.7
Cl1—Ir1—H1	88.4 (14)	C208—C209—H209	119.7
C107—P1—C101	104.0 (2)	C209—C210—C211	120.0 (6)
C107—P1—C2	104.7 (2)	C209—C210—H210	120.0
C101—P1—C2	103.4 (2)	C211—C210—H210	120.0
C107—P1—Ir1	117.25 (15)	C210—C211—C212	120.7 (6)
C101—P1—Ir1	119.28 (16)	C210—C211—H211	119.7
C2—P1—Ir1	106.46 (14)	C212—C211—H211	119.7
C1—P2—C201	116.7 (2)	C207—C212—C211	119.6 (6)
C1—P2—C207	114.7 (2)	C207—C212—H212	120.2
C201—P2—C207	104.9 (2)	C211—C212—H212	120.2
C1—P2—C2	106.8 (2)	C302—C301—C306	118.6 (4)
C201—P2—C2	108.4 (2)	C302—C301—P3	121.4 (4)
C207—P2—C2	104.6 (2)	C306—C301—P3	119.7 (4)
C1—P3—C301	120.2 (2)	C301—C302—C303	120.0 (5)
C1—P3—C307	114.8 (2)	C301—C302—H302	120.0
C301—P3—C307	103.7 (2)	C303—C302—H302	120.0
C1—P3—C3	106.6 (2)	C304—C303—C302	120.7 (5)
C301—P3—C3	101.9 (2)	C304—C303—H303	119.7
C307—P3—C3	108.6 (2)	C302—C303—H303	119.7
C401—P4—C407	101.6 (2)	C303—C304—C305	119.4 (5)
C401—P4—C3	104.2 (2)	C303—C304—H304	120.3
C407—P4—C3	103.4 (2)	C305—C304—H304	120.3
C401—P4—Ir1	118.52 (15)	C306—C305—C304	120.4 (5)
C407—P4—Ir1	120.92 (17)	C306—C305—H305	119.8
C3—P4—Ir1	106.10 (15)	C304—C305—H305	119.8
N1—C4—Ir1	175.0 (4)	C305—C306—C301	120.8 (5)
P3—C1—P2	127.8 (3)	C305—C306—H306	119.6
P3—C1—Ir1	119.7 (2)	C301—C306—H306	119.6
P2—C1—Ir1	112.5 (2)	C308—C307—C312	118.3 (5)
P2—C2—P1	105.9 (2)	C308—C307—P3	118.9 (4)
P2—C2—H2A	110.6	C312—C307—P3	122.7 (4)
P1—C2—H2A	110.6	C307—C308—C309	121.0 (5)
P2—C2—H2B	110.6	C307—C308—H308	119.5
P1—C2—H2B	110.6	C309—C308—H308	119.5
H2A—C2—H2B	108.7	C310—C309—C308	120.1 (6)
P3—C3—P4	110.8 (2)	C310—C309—H309	119.9
P3—C3—H3A	109.5	C308—C309—H309	119.9
P4—C3—H3A	109.5	C309—C310—C311	120.2 (6)
P3—C3—H3B	109.5	C309—C310—H310	119.9
P4—C3—H3B	109.5	C311—C310—H310	119.9
H3A—C3—H3B	108.1	C310—C311—C312	119.9 (6)
C102—C101—C106	119.0 (4)	C310—C311—H311	120.1
C102—C101—P1	118.7 (4)	C312—C311—H311	120.1
C106—C101—P1	122.2 (4)	C307—C312—C311	120.5 (5)
C103—C102—C101	119.8 (5)	C307—C312—H312	119.7
C103—C102—H102	120.1	C311—C312—H312	119.7
C101—C102—H102	120.1	C406—C401—C402	117.7 (5)
C104—C103—C102	120.8 (5)	C406—C401—P4	120.1 (4)
C104—C103—H103	119.6	C402—C401—P4	121.9 (4)
C102—C103—H103	119.6	C401—C402—C403	122.2 (6)
C105—C104—C103	119.9 (5)	C401—C402—H402	118.9
C105—C104—H104	120.1	C403—C402—H402	118.9
C103—C104—H104	120.1	C404—C403—C402	119.7 (7)
C104—C105—C106	120.8 (5)	C404—C403—H403	120.1
C104—C105—H105	119.6	C402—C403—H403	120.1
C106—C105—H105	119.6	C405—C404—C403	120.1 (6)
C105—C106—C101	119.6 (5)	C405—C404—H404	119.9
C105—C106—H106	120.2	C403—C404—H404	119.9
C101—C106—H106	120.2	C404—C405—C406	120.6 (6)
C108—C107—C112	117.8 (4)	C404—C405—H405	119.7
C108—C107—P1	119.9 (4)	C406—C405—H405	119.7
C112—C107—P1	122.3 (4)	C401—C406—C405	119.6 (6)
C109—C108—C107	121.5 (5)	C401—C406—H406	120.2
C109—C108—H108	119.2	C405—C406—H406	120.2
C107—C108—H108	119.2	C408—C407—C412	118.2 (5)
C110—C109—C108	119.8 (5)	C408—C407—P4	121.6 (4)
C110—C109—H109	120.1	C412—C407—P4	120.2 (4)
C108—C109—H109	120.1	C407—C408—C409	121.0 (5)
C109—C110—C111	120.0 (5)	C407—C408—H408	119.5
C109—C110—H110	120.0	C409—C408—H408	119.5
C111—C110—H110	120.0	C410—C409—C408	120.2 (6)
C110—C111—C112	120.5 (5)	C410—C409—H409	119.9
C110—C111—H111	119.7	C408—C409—H409	119.9
C112—C111—H111	119.7	C411—C410—C409	119.6 (6)
C111—C112—C107	120.2 (5)	C411—C410—H410	120.2
C111—C112—H112	119.9	C409—C410—H410	120.2
C107—C112—H112	119.9	C410—C411—C412	121.1 (6)
C202—C201—C206	118.6 (5)	C410—C411—H411	119.4
C202—C201—P2	122.0 (4)	C412—C411—H411	119.4
C206—C201—P2	119.4 (4)	C411—C412—C407	119.8 (6)
C201—C202—C203	120.5 (6)	C411—C412—H412	120.1
C201—C202—H202	119.7	C407—C412—H412	120.1
C203—C202—H202	119.7	N2—C5—C6	177.6 (10)
C204—C203—C202	119.9 (6)	C5—C6—H6A	109.5
C204—C203—H203	120.1	C5—C6—H6B	109.5
C202—C203—H203	120.1	H6A—C6—H6B	109.5
C203—C204—C205	120.8 (6)	C5—C6—H6C	109.5
C203—C204—H204	119.6	H6A—C6—H6C	109.5
C205—C204—H204	119.6	H6B—C6—H6C	109.5
			
C301—P3—C1—P2	76.6 (4)	C2—P2—C207—C212	42.9 (5)
C307—P3—C1—P2	−48.1 (4)	C1—P2—C207—C208	−21.3 (5)
C3—P3—C1—P2	−168.3 (3)	C201—P2—C207—C208	108.0 (4)
C301—P3—C1—Ir1	−101.9 (3)	C2—P2—C207—C208	−138.0 (4)
C307—P3—C1—Ir1	133.4 (2)	C212—C207—C208—C209	0.7 (8)
C3—P3—C1—Ir1	13.1 (3)	P2—C207—C208—C209	−178.4 (4)
C201—P2—C1—P3	−13.5 (4)	C207—C208—C209—C210	−1.7 (9)
C207—P2—C1—P3	109.7 (3)	C208—C209—C210—C211	1.9 (10)
C2—P2—C1—P3	−134.9 (3)	C209—C210—C211—C212	−1.2 (10)
C201—P2—C1—Ir1	165.1 (2)	C208—C207—C212—C211	−0.1 (8)
C207—P2—C1—Ir1	−71.7 (3)	P2—C207—C212—C211	179.0 (4)
C2—P2—C1—Ir1	43.7 (3)	C210—C211—C212—C207	0.3 (9)
C1—P2—C2—P1	−45.5 (3)	C1—P3—C301—C302	−1.3 (5)
C201—P2—C2—P1	−172.0 (2)	C307—P3—C301—C302	128.6 (4)
C207—P2—C2—P1	76.5 (3)	C3—P3—C301—C302	−118.7 (4)
C107—P1—C2—P2	−96.5 (2)	C1—P3—C301—C306	171.8 (4)
C101—P1—C2—P2	154.8 (2)	C307—P3—C301—C306	−58.3 (4)
Ir1—P1—C2—P2	28.3 (2)	C3—P3—C301—C306	54.4 (4)
C1—P3—C3—P4	13.2 (3)	C306—C301—C302—C303	3.4 (8)
C301—P3—C3—P4	140.1 (2)	P3—C301—C302—C303	176.6 (4)
C307—P3—C3—P4	−110.9 (3)	C301—C302—C303—C304	−1.0 (8)
C401—P4—C3—P3	−156.9 (2)	C302—C303—C304—C305	−1.4 (8)
C407—P4—C3—P3	97.2 (3)	C303—C304—C305—C306	1.3 (9)
Ir1—P4—C3—P3	−31.1 (3)	C304—C305—C306—C301	1.2 (8)
C107—P1—C101—C102	98.8 (4)	C302—C301—C306—C305	−3.6 (8)
C2—P1—C101—C102	−152.1 (4)	P3—C301—C306—C305	−176.9 (4)
Ir1—P1—C101—C102	−34.2 (4)	C1—P3—C307—C308	−65.0 (5)
C107—P1—C101—C106	−82.2 (4)	C301—P3—C307—C308	161.9 (4)
C2—P1—C101—C106	27.0 (4)	C3—P3—C307—C308	54.1 (5)
Ir1—P1—C101—C106	144.9 (3)	C1—P3—C307—C312	110.4 (4)
C106—C101—C102—C103	0.5 (7)	C301—P3—C307—C312	−22.6 (5)
P1—C101—C102—C103	179.6 (4)	C3—P3—C307—C312	−130.4 (4)
C101—C102—C103—C104	−1.2 (9)	C312—C307—C308—C309	−0.4 (9)
C102—C103—C104—C105	0.8 (9)	P3—C307—C308—C309	175.2 (5)
C103—C104—C105—C106	0.4 (9)	C307—C308—C309—C310	−0.3 (11)
C104—C105—C106—C101	−1.1 (8)	C308—C309—C310—C311	1.0 (12)
C102—C101—C106—C105	0.6 (7)	C309—C310—C311—C312	−1.0 (11)
P1—C101—C106—C105	−178.5 (4)	C308—C307—C312—C311	0.4 (8)
C101—P1—C107—C108	−121.7 (4)	P3—C307—C312—C311	−175.1 (5)
C2—P1—C107—C108	130.1 (4)	C310—C311—C312—C307	0.3 (10)
Ir1—P1—C107—C108	12.4 (4)	C407—P4—C401—C406	−38.3 (5)
C101—P1—C107—C112	59.7 (4)	C3—P4—C401—C406	−145.5 (4)
C2—P1—C107—C112	−48.5 (4)	Ir1—P4—C401—C406	96.9 (4)
Ir1—P1—C107—C112	−166.2 (3)	C407—P4—C401—C402	148.4 (4)
C112—C107—C108—C109	1.4 (7)	C3—P4—C401—C402	41.2 (5)
P1—C107—C108—C109	−177.2 (4)	Ir1—P4—C401—C402	−76.4 (4)
C107—C108—C109—C110	0.8 (8)	C406—C401—C402—C403	2.6 (8)
C108—C109—C110—C111	−2.8 (9)	P4—C401—C402—C403	176.1 (4)
C109—C110—C111—C112	2.5 (8)	C401—C402—C403—C404	−0.4 (9)
C110—C111—C112—C107	−0.2 (8)	C402—C403—C404—C405	−2.0 (10)
C108—C107—C112—C111	−1.7 (7)	C403—C404—C405—C406	2.0 (10)
P1—C107—C112—C111	176.9 (4)	C402—C401—C406—C405	−2.5 (8)
C1—P2—C201—C202	−84.0 (5)	P4—C401—C406—C405	−176.1 (4)
C207—P2—C201—C202	147.9 (4)	C404—C405—C406—C401	0.3 (9)
C2—P2—C201—C202	36.6 (5)	C401—P4—C407—C408	123.2 (5)
C1—P2—C201—C206	93.2 (5)	C3—P4—C407—C408	−129.0 (5)
C207—P2—C201—C206	−34.9 (5)	Ir1—P4—C407—C408	−10.6 (5)
C2—P2—C201—C206	−146.2 (4)	C401—P4—C407—C412	−57.8 (5)
C206—C201—C202—C203	−1.8 (8)	C3—P4—C407—C412	50.1 (5)
P2—C201—C202—C203	175.4 (4)	Ir1—P4—C407—C412	168.5 (4)
C201—C202—C203—C204	0.4 (9)	C412—C407—C408—C409	−0.1 (9)
C202—C203—C204—C205	1.1 (10)	P4—C407—C408—C409	179.0 (5)
C203—C204—C205—C206	−1.1 (10)	C407—C408—C409—C410	−1.7 (11)
C202—C201—C206—C205	1.7 (8)	C408—C409—C410—C411	2.1 (12)
P2—C201—C206—C205	−175.6 (5)	C409—C410—C411—C412	−0.6 (13)
C204—C205—C206—C201	−0.2 (9)	C410—C411—C412—C407	−1.2 (11)
C1—P2—C207—C212	159.6 (4)	C408—C407—C412—C411	1.5 (9)
C201—P2—C207—C212	−71.0 (5)	P4—C407—C412—C411	−177.6 (5)

### Chloridocyanidohydrido(1,1,3,3,5,5,7,7-octaphenyl-1,3λ5,5λ4,7-tetraphospha-κ2P1,P7-hept-3-en-4-yl)iridium(III) acetonitrile monosolvate (1b) Hydrogen-bond geometry (Å, º)

**Table:**

*D*—H···*A*	*D*—H	H···*A*	*D*···*A*	*D*—H···*A*
C3—H3*B*···N2	0.98	2.58	3.4925 (1)	156
C102—H102···Cl1	0.94	2.66	3.5216 (1)	153
C112—H112···Cl1^i^	0.94	2.77	3.6470 (1)	155
C210—H210···N2^ii^	0.94	2.61	3.4554 (1)	150
C303—H303···N1^iii^	0.94	2.48	3.2064 (1)	134

Symmetry codes: (i) −*x*+5/2, *y*+1/2, −*z*+3/2; (ii) *x*+1/2, −*y*+1/2, *z*+1/2; (iii) −*x*+2, −*y*+1, −*z*+1.

### [7-(4-Chlorophenyl)-1,1,3,3-tetraphenyl-5,6,7-triaza-κN7-1,3λ4-diphospha-κP1-hepta-4,6-dien-4-yl][methylenebis(diphenylphosphine)-κ2P,P']iridium(I) chloride–dichloromethane–toluene (2/3/1) (2) Crystal data

**Table:**

[Ir(C_25_H_22_P_2_)(C_32_H_26_ClN_3_P_2_)]Cl·0.5C_7_H_8_·1.5CH_2_Cl_2_	*D*_x_ = 1.397 Mg m^−^^3^
*M**_r_* = 1335.42	Mo *K*α radiation, λ = 0.71073 Å
Orthorhombic, *P**b**c**n*	Cell parameters from 221720 reflections
*a* = 28.1283 (3) Å	θ = 1.0–26.0°
*b* = 19.0989 (2) Å	µ = 2.45 mm^−^^1^
*c* = 23.6339 (2) Å	*T* = 233 K
*V* = 12696.6 (2) Å^3^	Prism, red-brown
*Z* = 8	0.3 × 0.2 × 0.15 mm
*F*(000) = 5360	

### [7-(4-Chlorophenyl)-1,1,3,3-tetraphenyl-5,6,7-triaza-κN7-1,3λ4-diphospha-κP1-hepta-4,6-dien-4-yl][methylenebis(diphenylphosphine)-κ2P,P']iridium(I) chloride–dichloromethane–toluene (2/3/1) (2) Data collection

**Table:**

Nonius KappaCCD diffractometer	*R*_int_ = 0.039
phi– and ω–scans	θ_max_ = 25.0°, θ_min_ = 1.9°
73594 measured reflections	*h* = −33→33
11155 independent reflections	*k* = −22→22
9002 reflections with *I* > 2σ(*I*)	*l* = −19→28

### [7-(4-Chlorophenyl)-1,1,3,3-tetraphenyl-5,6,7-triaza-κN7-1,3λ4-diphospha-κP1-hepta-4,6-dien-4-yl][methylenebis(diphenylphosphine)-κ2P,P']iridium(I) chloride–dichloromethane–toluene (2/3/1) (2) Refinement

**Table:**

Refinement on *F*^2^	30 restraints
Least-squares matrix: full	Hydrogen site location: inferred from neighbouring sites
*R*[*F*^2^ > 2σ(*F*^2^)] = 0.042	H-atom parameters constrained
*wR*(*F*^2^) = 0.126	*w* = 1/[σ^2^(*F*_o_^2^) + (0.0615*P*)^2^ + 44.6168*P*] where *P* = (*F*_o_^2^ + 2*F*_c_^2^)/3
*S* = 1.05	(Δ/σ)_max_ = 0.002
11155 reflections	Δρ_max_ = 1.66 e Å^−^^3^
724 parameters	Δρ_min_ = −0.73 e Å^−^^3^

### [7-(4-Chlorophenyl)-1,1,3,3-tetraphenyl-5,6,7-triaza-κN7-1,3λ4-diphospha-κP1-hepta-4,6-dien-4-yl][methylenebis(diphenylphosphine)-κ2P,P']iridium(I) chloride–dichloromethane–toluene (2/3/1) (2) Special details

**Table:**

Experimental. All data sets were measured with several scans to increase the number of redundant reflections. In our experience this method of averaging redundant reflections replaces in a good approximation semi-empirical absorption methods (absorption correction programs like SORTAV lead to no better data sets).
Geometry. All esds (except the esd in the dihedral angle between two l.s. planes) are estimated using the full covariance matrix. The cell esds are taken into account individually in the estimation of esds in distances, angles and torsion angles; correlations between esds in cell parameters are only used when they are defined by crystal symmetry. An approximate (isotropic) treatment of cell esds is used for estimating esds involving l.s. planes.
Refinement. 2:1 positional disorder of the counter anion Cl1:Cl1A. The solvent molecules CH_2_Cl_2_ and C_7_H_8_ show occupational disorder, whereas the toluene shows additional positional disorder of ratio 1:1 with some nearly overlying carbon atoms. We think, because of short intermolecular Cl···C contacts between CH_2_Cl_2_ and C_7_H_8_, that there is a correlation between these molecules. Therefore, the two solvate molecules Cl3—C10–l4 and Cl5—C11—Cl6 have an occupancy of 0.75 and the `two' toluene molecules, C12–C18 and C19–C25, an occupancy of 0.25. Several bond restraints must be used to refine the toluene carbon atoms reasonably isotropically.

### [7-(4-Chlorophenyl)-1,1,3,3-tetraphenyl-5,6,7-triaza-κN7-1,3λ4-diphospha-κP1-hepta-4,6-dien-4-yl][methylenebis(diphenylphosphine)-κ2P,P']iridium(I) chloride–dichloromethane–toluene (2/3/1) (2) Fractional atomic coordinates and isotropic or equivalent isotropic displacement parameters (Å^2^)

**Table:**

	*x*	*y*	*z*	*U*_iso_*/*U*_eq_	Occ. (<1)
Ir1	0.37466 (2)	0.64909 (2)	−0.05631 (2)	0.03621 (9)	
P1	0.32978 (5)	0.71589 (7)	−0.11572 (6)	0.0422 (3)	
P2	0.35994 (5)	0.59865 (8)	−0.19050 (6)	0.0410 (3)	
P3	0.36065 (6)	0.70441 (8)	0.03213 (6)	0.0451 (3)	
P4	0.31226 (5)	0.59668 (8)	−0.01288 (6)	0.0437 (3)	
Cl1	0.26332 (16)	0.71646 (19)	−0.30485 (14)	0.0456 (8)	0.65
Cl1A	0.2678 (3)	0.7368 (3)	−0.3219 (2)	0.0476 (18)	0.35
Cl2	0.57143 (7)	0.68950 (13)	0.14255 (9)	0.0891 (6)	
N1	0.44176 (16)	0.6172 (2)	−0.04574 (17)	0.0402 (10)	
N2	0.46127 (17)	0.5721 (3)	−0.08336 (19)	0.0470 (11)	
N3	0.43390 (16)	0.5618 (2)	−0.12734 (19)	0.0454 (11)	
C1	0.3915 (2)	0.5945 (3)	−0.1255 (2)	0.0405 (12)	
C2	0.3129 (2)	0.6596 (3)	−0.1760 (2)	0.0431 (12)	
H2A	0.2837	0.6341	−0.1670	0.052*	
H2B	0.3070	0.6886	−0.2095	0.052*	
C3	0.3010 (2)	0.6647 (3)	0.0405 (3)	0.0524 (14)	
H3A	0.2956	0.6456	0.0784	0.063*	
H3B	0.2752	0.6965	0.0299	0.063*	
C4	0.47418 (19)	0.6336 (3)	−0.0010 (2)	0.0421 (12)	
C5	0.4929 (2)	0.6995 (3)	0.0022 (3)	0.0575 (15)	
H5	0.4846	0.7332	−0.0250	0.069*	
C6	0.5240 (2)	0.7170 (4)	0.0453 (3)	0.0654 (18)	
H6	0.5373	0.7622	0.0474	0.078*	
C7	0.5352 (2)	0.6665 (4)	0.0852 (3)	0.0607 (17)	
C8	0.5178 (2)	0.6007 (4)	0.0822 (3)	0.0576 (16)	
H8	0.5266	0.5669	0.1091	0.069*	
C9	0.4868 (2)	0.5838 (3)	0.0388 (3)	0.0539 (15)	
H9	0.4743	0.5383	0.0364	0.065*	
C101	0.2731 (2)	0.7563 (3)	−0.0966 (2)	0.0506 (14)	
C102	0.2743 (3)	0.8171 (4)	−0.0636 (3)	0.0679 (19)	
H102	0.3034	0.8384	−0.0545	0.081*	
C103	0.2310 (4)	0.8460 (5)	−0.0439 (3)	0.084 (3)	
H103	0.2312	0.8864	−0.0211	0.101*	
C104	0.1885 (4)	0.8149 (6)	−0.0582 (4)	0.094 (3)	
H104	0.1599	0.8339	−0.0445	0.113*	
C105	0.1871 (3)	0.7572 (5)	−0.0918 (4)	0.090 (3)	
H105	0.1578	0.7374	−0.1024	0.108*	
C106	0.2295 (2)	0.7277 (4)	−0.1104 (3)	0.0679 (18)	
H106	0.2284	0.6872	−0.1330	0.081*	
C107	0.3610 (2)	0.7881 (3)	−0.1498 (2)	0.0525 (15)	
C108	0.4091 (3)	0.7953 (4)	−0.1443 (3)	0.073 (2)	
H108	0.4261	0.7643	−0.1209	0.088*	
C109	0.4331 (4)	0.8475 (5)	−0.1729 (5)	0.107 (3)	
H109	0.4661	0.8523	−0.1685	0.129*	
C110	0.4091 (5)	0.8922 (5)	−0.2075 (5)	0.113 (4)	
H110	0.4255	0.9278	−0.2267	0.136*	
C111	0.3607 (4)	0.8854 (4)	−0.2143 (4)	0.098 (3)	
H111	0.3442	0.9157	−0.2388	0.117*	
C112	0.3369 (3)	0.8348 (4)	−0.1857 (3)	0.070 (2)	
H112	0.3038	0.8309	−0.1900	0.084*	
C201	0.3333 (2)	0.5177 (3)	−0.2137 (2)	0.0485 (13)	
C202	0.3455 (2)	0.4540 (3)	−0.1901 (3)	0.0620 (17)	
H202	0.3684	0.4521	−0.1611	0.074*	
C203	0.3240 (3)	0.3927 (4)	−0.2092 (4)	0.079 (2)	
H203	0.3326	0.3494	−0.1932	0.095*	
C204	0.2907 (3)	0.3951 (4)	−0.2508 (3)	0.076 (2)	
H204	0.2764	0.3535	−0.2637	0.091*	
C205	0.2776 (3)	0.4592 (5)	−0.2743 (3)	0.081 (2)	
H205	0.2543	0.4607	−0.3027	0.097*	
C206	0.2988 (2)	0.5206 (4)	−0.2560 (3)	0.0653 (17)	
H206	0.2900	0.5638	−0.2720	0.078*	
C207	0.3977 (2)	0.6277 (3)	−0.2470 (2)	0.0485 (13)	
C208	0.4304 (3)	0.5804 (4)	−0.2676 (3)	0.0675 (18)	
H208	0.4314	0.5345	−0.2531	0.081*	
C209	0.4618 (3)	0.6005 (5)	−0.3096 (3)	0.084 (2)	
H209	0.4831	0.5674	−0.3245	0.101*	
C210	0.4627 (3)	0.6665 (5)	−0.3295 (3)	0.080 (2)	
H210	0.4855	0.6798	−0.3565	0.096*	
C211	0.4296 (4)	0.7149 (5)	−0.3102 (3)	0.090 (3)	
H211	0.4295	0.7608	−0.3246	0.108*	
C212	0.3961 (3)	0.6950 (4)	−0.2688 (3)	0.071 (2)	
H212	0.3730	0.7269	−0.2561	0.085*	
C301	0.3552 (2)	0.7980 (3)	0.0390 (3)	0.0555 (15)	
C302	0.3843 (3)	0.8405 (4)	0.0069 (4)	0.073 (2)	
H302	0.4062	0.8204	−0.0185	0.087*	
C303	0.3814 (4)	0.9136 (5)	0.0121 (5)	0.108 (3)	
H303	0.4009	0.9430	−0.0098	0.130*	
C304	0.3495 (5)	0.9411 (5)	0.0500 (6)	0.119 (4)	
H304	0.3472	0.9900	0.0536	0.143*	
C305	0.3215 (4)	0.9003 (5)	0.0819 (4)	0.099 (3)	
H305	0.3004	0.9211	0.1078	0.119*	
C306	0.3230 (3)	0.8279 (4)	0.0774 (3)	0.072 (2)	
H306	0.3030	0.7995	0.0995	0.087*	
C307	0.3913 (2)	0.6819 (4)	0.0979 (2)	0.0531 (15)	
C308	0.4215 (3)	0.7297 (5)	0.1240 (3)	0.076 (2)	
H308	0.4272	0.7732	0.1068	0.092*	
C309	0.4432 (3)	0.7140 (6)	0.1749 (4)	0.090 (3)	
H309	0.4627	0.7472	0.1928	0.108*	
C310	0.4358 (3)	0.6490 (6)	0.1991 (3)	0.097 (3)	
H310	0.4511	0.6376	0.2333	0.116*	
C311	0.4066 (4)	0.6015 (6)	0.1741 (4)	0.107 (3)	
H311	0.4013	0.5577	0.1912	0.129*	
C312	0.3845 (3)	0.6181 (5)	0.1232 (3)	0.086 (2)	
H312	0.3646	0.5849	0.1058	0.103*	
C401	0.2564 (2)	0.5722 (3)	−0.0459 (2)	0.0507 (14)	
C402	0.2583 (2)	0.5251 (4)	−0.0908 (3)	0.0604 (16)	
H402	0.2879	0.5079	−0.1032	0.072*	
C403	0.2169 (3)	0.5034 (4)	−0.1173 (3)	0.077 (2)	
H403	0.2183	0.4712	−0.1474	0.093*	
C404	0.1747 (3)	0.5286 (5)	−0.1000 (4)	0.091 (3)	
H404	0.1468	0.5146	−0.1189	0.109*	
C405	0.1716 (3)	0.5739 (5)	−0.0558 (4)	0.091 (3)	
H405	0.1417	0.5903	−0.0440	0.109*	
C406	0.2126 (2)	0.5962 (4)	−0.0277 (3)	0.074 (2)	
H406	0.2105	0.6271	0.0032	0.089*	
C407	0.3282 (2)	0.5166 (3)	0.0241 (2)	0.0494 (14)	
C408	0.3714 (2)	0.4846 (3)	0.0137 (3)	0.0570 (16)	
H408	0.3917	0.5024	−0.0145	0.068*	
C409	0.3848 (3)	0.4258 (4)	0.0449 (3)	0.071 (2)	
H409	0.4143	0.4040	0.0383	0.085*	
C410	0.3547 (3)	0.4002 (4)	0.0855 (3)	0.076 (2)	
H410	0.3642	0.3616	0.1073	0.091*	
C411	0.3110 (3)	0.4297 (4)	0.0948 (3)	0.075 (2)	
H411	0.2904	0.4104	0.1219	0.089*	
C412	0.2976 (3)	0.4877 (4)	0.0645 (3)	0.0638 (18)	
H412	0.2677	0.5080	0.0708	0.077*	
C12	0.6027 (14)	0.889 (2)	−0.113 (2)	0.150 (19)*	0.25
C13	0.5711 (18)	0.867 (2)	−0.069 (2)	0.18 (3)*	0.25
H13	0.5786	0.8275	−0.0469	0.216*	0.25
C14	0.5296 (18)	0.903 (3)	−0.059 (2)	0.19 (3)*	0.25
H14	0.5080	0.8870	−0.0314	0.227*	0.25
C15	0.5195 (13)	0.964 (2)	−0.0888 (16)	0.112 (13)*	0.25
H15	0.4965	0.9958	−0.0758	0.134*	0.25
C16	0.5445 (13)	0.9764 (19)	−0.1389 (15)	0.098 (11)*	0.25
H16	0.5334	1.0100	−0.1648	0.118*	0.25
C17	0.585 (2)	0.940 (4)	−0.150 (2)	0.27 (5)*	0.25
H17	0.6018	0.9488	−0.1839	0.330*	0.25
C18	0.648 (2)	0.844 (4)	−0.127 (4)	0.22 (5)*	0.25
H18A	0.6544	0.8116	−0.0963	0.325*	0.25
H18B	0.6424	0.8172	−0.1616	0.325*	0.25
H18C	0.6751	0.8743	−0.1325	0.325*	0.25
C19	0.5276 (16)	0.991 (2)	−0.1188 (19)	0.141 (18)*	0.25
C20	0.491 (2)	1.019 (3)	−0.0841 (19)	0.22 (3)*	0.25
H20	0.4846	0.9993	−0.0487	0.262*	0.25
C21	0.4658 (11)	1.0766 (17)	−0.1020 (14)	0.092 (10)*	0.25
H21	0.4423	1.0959	−0.0784	0.111*	0.25
C22	0.4743 (15)	1.106 (2)	−0.1540 (17)	0.149 (18)*	0.25
H22	0.4534	1.1396	−0.1687	0.178*	0.25
C23	0.5139 (16)	1.085 (2)	−0.1849 (15)	0.152 (19)*	0.25
H23	0.5211	1.1076	−0.2194	0.183*	0.25
C24	0.5423 (15)	1.033 (3)	−0.165 (2)	0.18 (2)*	0.25
H24	0.5720	1.0251	−0.1820	0.215*	0.25
C25	0.555 (2)	0.922 (2)	−0.103 (3)	0.18 (2)*	0.25
H25A	0.5322	0.8853	−0.0952	0.269*	0.25
H25B	0.5740	0.9307	−0.0694	0.269*	0.25
H25C	0.5753	0.9087	−0.1340	0.269*	0.25
C10	0.6644 (7)	0.7998 (10)	−0.1009 (12)	0.182 (11)	0.75
H10A	0.6573	0.7705	−0.1339	0.218*	0.75
H10B	0.6925	0.7800	−0.0824	0.218*	0.75
Cl3	0.6794 (3)	0.8884 (3)	−0.1262 (2)	0.194 (3)	0.75
Cl4	0.6209 (3)	0.7945 (4)	−0.0581 (2)	0.180 (2)	0.75
C11	0.3470 (6)	1.1480 (6)	−0.2714 (6)	0.105 (4)	0.75
H11A	0.3661	1.1584	−0.3050	0.126*	0.75
H11B	0.3161	1.1707	−0.2764	0.126*	0.75
Cl5	0.3737 (2)	1.1831 (3)	−0.2153 (2)	0.178 (2)	0.75
Cl6	0.3386 (3)	1.0614 (2)	−0.2681 (2)	0.180 (3)	0.75

### [7-(4-Chlorophenyl)-1,1,3,3-tetraphenyl-5,6,7-triaza-κN7-1,3λ4-diphospha-κP1-hepta-4,6-dien-4-yl][methylenebis(diphenylphosphine)-κ2P,P']iridium(I) chloride–dichloromethane–toluene (2/3/1) (2) Atomic displacement parameters (Å^2^)

**Table:**

	*U*^11^	*U*^22^	*U*^33^	*U*^12^	*U*^13^	*U*^23^
Ir1	0.04080 (14)	0.03873 (13)	0.02908 (13)	0.00223 (8)	0.00040 (8)	0.00056 (8)
P1	0.0501 (8)	0.0421 (7)	0.0342 (7)	0.0083 (6)	−0.0006 (6)	0.0002 (6)
P2	0.0471 (8)	0.0430 (7)	0.0328 (7)	0.0031 (6)	0.0002 (6)	−0.0040 (6)
P3	0.0513 (8)	0.0505 (8)	0.0336 (7)	0.0017 (7)	0.0012 (6)	−0.0051 (6)
P4	0.0449 (8)	0.0498 (8)	0.0365 (7)	−0.0025 (6)	0.0018 (6)	0.0023 (6)
Cl1	0.0656 (15)	0.0489 (19)	0.0225 (18)	0.0087 (14)	−0.0159 (15)	0.0030 (12)
Cl1A	0.080 (4)	0.053 (4)	0.010 (3)	0.041 (3)	−0.025 (3)	−0.008 (2)
Cl2	0.0687 (12)	0.1216 (18)	0.0771 (12)	−0.0142 (11)	−0.0299 (10)	−0.0132 (12)
N1	0.041 (2)	0.045 (3)	0.034 (2)	0.000 (2)	0.0021 (19)	0.0033 (19)
N2	0.047 (3)	0.054 (3)	0.040 (3)	0.008 (2)	0.002 (2)	0.001 (2)
N3	0.047 (3)	0.049 (3)	0.041 (3)	0.004 (2)	−0.001 (2)	−0.001 (2)
C1	0.048 (3)	0.038 (3)	0.035 (3)	0.002 (2)	0.002 (2)	0.000 (2)
C2	0.046 (3)	0.049 (3)	0.034 (3)	0.005 (2)	−0.003 (2)	0.000 (2)
C3	0.055 (4)	0.062 (4)	0.040 (3)	0.001 (3)	0.005 (3)	−0.003 (3)
C4	0.038 (3)	0.049 (3)	0.039 (3)	−0.002 (2)	0.002 (2)	0.002 (2)
C5	0.060 (4)	0.057 (4)	0.056 (4)	−0.004 (3)	−0.004 (3)	0.007 (3)
C6	0.064 (4)	0.062 (4)	0.070 (4)	−0.022 (3)	−0.011 (3)	0.000 (3)
C7	0.042 (3)	0.087 (5)	0.053 (4)	−0.002 (3)	−0.008 (3)	−0.013 (4)
C8	0.059 (4)	0.064 (4)	0.050 (3)	0.006 (3)	−0.013 (3)	0.002 (3)
C9	0.065 (4)	0.050 (3)	0.046 (3)	−0.003 (3)	−0.009 (3)	0.003 (3)
C101	0.058 (4)	0.054 (3)	0.040 (3)	0.019 (3)	0.001 (3)	0.001 (3)
C102	0.080 (5)	0.070 (5)	0.054 (4)	0.027 (4)	−0.010 (3)	−0.013 (3)
C103	0.104 (7)	0.088 (6)	0.060 (4)	0.049 (5)	−0.001 (4)	−0.020 (4)
C104	0.084 (6)	0.110 (7)	0.089 (6)	0.049 (6)	0.015 (5)	0.007 (5)
C105	0.064 (5)	0.091 (6)	0.115 (7)	0.021 (4)	0.006 (5)	−0.011 (5)
C106	0.057 (4)	0.075 (5)	0.072 (4)	0.016 (3)	−0.001 (3)	−0.009 (4)
C107	0.070 (4)	0.045 (3)	0.043 (3)	0.003 (3)	−0.002 (3)	0.003 (3)
C108	0.081 (5)	0.070 (5)	0.069 (5)	−0.010 (4)	0.000 (4)	0.023 (4)
C109	0.100 (7)	0.107 (7)	0.114 (8)	−0.035 (6)	0.003 (6)	0.034 (6)
C110	0.146 (10)	0.079 (6)	0.115 (8)	−0.025 (6)	0.024 (7)	0.044 (6)
C111	0.160 (10)	0.060 (5)	0.073 (5)	0.008 (6)	0.021 (6)	0.028 (4)
C112	0.098 (6)	0.053 (4)	0.058 (4)	0.013 (4)	0.002 (4)	0.005 (3)
C201	0.052 (3)	0.051 (3)	0.042 (3)	−0.001 (3)	0.003 (3)	−0.003 (3)
C202	0.066 (4)	0.053 (4)	0.067 (4)	0.003 (3)	−0.009 (3)	−0.007 (3)
C203	0.090 (6)	0.054 (4)	0.093 (6)	−0.005 (4)	−0.008 (5)	−0.012 (4)
C204	0.078 (5)	0.067 (5)	0.082 (5)	−0.017 (4)	−0.004 (4)	−0.015 (4)
C205	0.076 (5)	0.094 (6)	0.072 (5)	−0.017 (4)	−0.021 (4)	−0.013 (4)
C206	0.070 (4)	0.068 (4)	0.057 (4)	−0.009 (3)	−0.013 (3)	−0.004 (3)
C207	0.055 (3)	0.059 (3)	0.032 (3)	0.000 (3)	−0.001 (3)	−0.009 (3)
C208	0.076 (5)	0.070 (4)	0.056 (4)	0.013 (4)	0.016 (3)	0.001 (3)
C209	0.072 (5)	0.122 (7)	0.058 (4)	0.022 (5)	0.026 (4)	0.009 (5)
C210	0.069 (5)	0.113 (7)	0.058 (4)	−0.013 (5)	0.019 (4)	−0.004 (5)
C211	0.127 (8)	0.082 (5)	0.061 (5)	−0.026 (5)	0.021 (5)	0.012 (4)
C212	0.094 (5)	0.062 (4)	0.057 (4)	−0.003 (4)	0.026 (4)	−0.004 (3)
C301	0.061 (4)	0.057 (4)	0.048 (3)	0.005 (3)	−0.004 (3)	−0.010 (3)
C302	0.086 (5)	0.057 (4)	0.075 (5)	0.001 (4)	−0.003 (4)	−0.003 (4)
C303	0.136 (9)	0.051 (5)	0.138 (9)	−0.010 (5)	−0.013 (7)	−0.002 (6)
C304	0.158 (11)	0.053 (5)	0.146 (10)	0.027 (7)	−0.038 (9)	−0.025 (6)
C305	0.120 (8)	0.083 (6)	0.096 (7)	0.039 (6)	−0.016 (6)	−0.039 (6)
C306	0.079 (5)	0.074 (5)	0.064 (4)	0.021 (4)	−0.009 (4)	−0.022 (4)
C307	0.056 (3)	0.070 (4)	0.034 (3)	0.003 (3)	−0.001 (3)	−0.003 (3)
C308	0.066 (4)	0.096 (6)	0.067 (5)	0.000 (4)	−0.012 (4)	−0.003 (4)
C309	0.067 (5)	0.133 (8)	0.070 (5)	−0.004 (5)	−0.023 (4)	−0.021 (5)
C310	0.084 (6)	0.164 (10)	0.042 (4)	0.009 (6)	−0.016 (4)	0.007 (5)
C311	0.130 (8)	0.131 (8)	0.060 (5)	−0.017 (7)	−0.026 (5)	0.038 (5)
C312	0.111 (7)	0.095 (6)	0.051 (4)	−0.020 (5)	−0.022 (4)	0.019 (4)
C401	0.047 (3)	0.056 (4)	0.049 (3)	−0.007 (3)	−0.003 (3)	0.007 (3)
C402	0.061 (4)	0.064 (4)	0.056 (4)	−0.012 (3)	0.000 (3)	−0.006 (3)
C403	0.073 (5)	0.089 (5)	0.070 (5)	−0.020 (4)	−0.011 (4)	−0.011 (4)
C404	0.074 (6)	0.122 (7)	0.076 (5)	−0.024 (5)	−0.021 (4)	0.002 (5)
C405	0.054 (5)	0.121 (8)	0.098 (7)	−0.005 (5)	−0.003 (4)	−0.003 (6)
C406	0.052 (4)	0.097 (6)	0.074 (5)	0.003 (4)	−0.004 (3)	−0.007 (4)
C407	0.059 (4)	0.050 (3)	0.039 (3)	−0.012 (3)	−0.005 (3)	0.003 (3)
C408	0.057 (4)	0.055 (4)	0.059 (4)	−0.007 (3)	−0.001 (3)	0.011 (3)
C409	0.079 (5)	0.048 (4)	0.085 (5)	0.002 (3)	−0.008 (4)	0.016 (4)
C410	0.109 (6)	0.048 (4)	0.070 (5)	−0.011 (4)	−0.021 (5)	0.017 (4)
C411	0.099 (6)	0.075 (5)	0.049 (4)	−0.025 (4)	0.000 (4)	0.021 (4)
C412	0.068 (4)	0.071 (4)	0.052 (4)	−0.012 (4)	0.004 (3)	0.006 (3)
C10	0.143 (15)	0.123 (15)	0.28 (3)	−0.022 (13)	0.029 (19)	−0.125 (18)
Cl3	0.282 (8)	0.178 (5)	0.121 (4)	−0.029 (6)	0.024 (4)	−0.015 (4)
Cl4	0.224 (7)	0.181 (6)	0.135 (4)	−0.024 (5)	0.009 (4)	−0.033 (4)
C11	0.120 (11)	0.088 (9)	0.106 (10)	0.001 (7)	−0.036 (9)	−0.005 (7)
Cl5	0.249 (7)	0.173 (5)	0.112 (3)	−0.071 (4)	−0.039 (3)	−0.028 (3)
Cl6	0.315 (8)	0.076 (2)	0.150 (4)	−0.040 (3)	−0.071 (4)	0.026 (2)

### [7-(4-Chlorophenyl)-1,1,3,3-tetraphenyl-5,6,7-triaza-κN7-1,3λ4-diphospha-κP1-hepta-4,6-dien-4-yl][methylenebis(diphenylphosphine)-κ2P,P']iridium(I) chloride–dichloromethane–toluene (2/3/1) (2) Geometric parameters (Å, º)

**Table:**

Ir1—P1	2.2788 (14)	C301—C302	1.380 (10)
Ir1—C1	1.998 (5)	C301—C306	1.402 (10)
Ir1—N1	1.999 (4)	C302—C303	1.405 (11)
Ir1—P3	2.3748 (14)	C302—H302	0.9400
Ir1—P4	2.2662 (14)	C303—C304	1.372 (16)
P1—C107	1.821 (6)	C303—H303	0.9400
P1—C101	1.827 (6)	C304—C305	1.341 (15)
P1—C2	1.846 (5)	C304—H304	0.9400
P2—C1	1.775 (5)	C305—C306	1.387 (12)
P2—C207	1.794 (6)	C305—H305	0.9400
P2—C2	1.795 (5)	C306—H306	0.9400
P2—C201	1.803 (6)	C307—C312	1.372 (11)
P3—C301	1.802 (7)	C307—C308	1.391 (10)
P3—C307	1.828 (6)	C308—C309	1.381 (11)
P3—C3	1.851 (6)	C308—H308	0.9400
P3—P4	2.686 (2)	C309—C310	1.384 (13)
P4—C401	1.817 (6)	C309—H309	0.9400
P4—C407	1.818 (6)	C310—C311	1.359 (13)
P4—C3	1.838 (6)	C310—H310	0.9400
Cl1—Cl1A	0.574 (6)	C311—C312	1.392 (11)
Cl2—C7	1.751 (6)	C311—H311	0.9400
N1—N2	1.354 (6)	C312—H312	0.9400
N1—C4	1.431 (7)	C401—C406	1.382 (9)
N2—N3	1.308 (6)	C401—C402	1.392 (9)
N3—C1	1.346 (7)	C402—C403	1.387 (9)
C2—H2A	0.9800	C402—H402	0.9400
C2—H2B	0.9800	C403—C404	1.345 (12)
C3—H3A	0.9800	C403—H403	0.9400
C3—H3B	0.9800	C404—C405	1.361 (12)
C4—C5	1.367 (8)	C404—H404	0.9400
C4—C9	1.384 (8)	C405—C406	1.398 (11)
C5—C6	1.383 (9)	C405—H405	0.9400
C5—H5	0.9400	C406—H406	0.9400
C6—C7	1.387 (10)	C407—C408	1.383 (9)
C6—H6	0.9400	C407—C412	1.398 (9)
C7—C8	1.350 (9)	C408—C409	1.396 (9)
C8—C9	1.384 (8)	C408—H408	0.9400
C8—H8	0.9400	C409—C410	1.369 (11)
C9—H9	0.9400	C409—H409	0.9400
C101—C106	1.384 (9)	C410—C411	1.369 (11)
C101—C102	1.400 (9)	C410—H410	0.9400
C102—C103	1.417 (11)	C411—C412	1.371 (10)
C102—H102	0.9400	C411—H411	0.9400
C103—C104	1.377 (13)	C412—H412	0.9400
C103—H103	0.9400	C12—C17	1.401 (17)
C104—C105	1.359 (13)	C12—C13	1.430 (17)
C104—H104	0.9400	C12—C18	1.575 (17)
C105—C106	1.389 (10)	C13—C14	1.381 (17)
C105—H105	0.9400	C13—H13	0.9400
C106—H106	0.9400	C14—C15	1.388 (17)
C107—C108	1.368 (10)	C14—H14	0.9400
C107—C112	1.406 (9)	C15—C16	1.397 (17)
C108—C109	1.381 (11)	C15—H15	0.9400
C108—H108	0.9400	C16—C17	1.369 (17)
C109—C110	1.360 (14)	C16—H16	0.9400
C109—H109	0.9400	C17—H17	0.9400
C110—C111	1.375 (14)	C18—H18A	0.9700
C110—H110	0.9400	C18—H18B	0.9700
C111—C112	1.356 (11)	C18—H18C	0.9700
C111—H111	0.9400	C19—C24	1.421 (17)
C112—H112	0.9400	C19—C20	1.422 (17)
C201—C202	1.382 (9)	C19—C25	1.559 (16)
C201—C206	1.395 (9)	C20—C21	1.373 (16)
C202—C203	1.394 (9)	C20—H20	0.9400
C202—H202	0.9400	C21—C22	1.370 (17)
C203—C204	1.361 (11)	C21—H21	0.9400
C203—H203	0.9400	C22—C23	1.387 (17)
C204—C205	1.392 (11)	C22—H22	0.9400
C204—H204	0.9400	C23—C24	1.362 (17)
C205—C206	1.385 (10)	C23—H23	0.9400
C205—H205	0.9400	C24—H24	0.9400
C206—H206	0.9400	C25—H25A	0.9700
C207—C208	1.380 (9)	C25—H25B	0.9700
C207—C212	1.384 (9)	C25—H25C	0.9700
C208—C209	1.382 (10)	C10—Cl4	1.59 (2)
C208—H208	0.9400	C10—Cl3	1.84 (2)
C209—C210	1.347 (12)	C10—H10A	0.9800
C209—H209	0.9400	C10—H10B	0.9800
C210—C211	1.389 (12)	C11—Cl5	1.665 (13)
C210—H210	0.9400	C11—Cl6	1.672 (12)
C211—C212	1.411 (10)	C11—H11A	0.9800
C211—H211	0.9400	C11—H11B	0.9800
C212—H212	0.9400		
			
C1—Ir1—N1	73.7 (2)	C208—C209—H209	119.2
C1—Ir1—P4	108.92 (16)	C209—C210—C211	119.7 (7)
N1—Ir1—P4	122.71 (13)	C209—C210—H210	120.1
C1—Ir1—P1	85.35 (15)	C211—C210—H210	120.1
N1—Ir1—P1	140.40 (13)	C210—C211—C212	119.7 (8)
P4—Ir1—P1	95.59 (5)	C210—C211—H211	120.1
C1—Ir1—P3	173.04 (16)	C212—C211—H211	120.1
N1—Ir1—P3	100.49 (13)	C207—C212—C211	119.1 (7)
P4—Ir1—P3	70.69 (5)	C207—C212—H212	120.4
P1—Ir1—P3	101.62 (5)	C211—C212—H212	120.4
C107—P1—C101	102.1 (3)	C302—C301—C306	120.0 (7)
C107—P1—C2	102.9 (3)	C302—C301—P3	118.8 (5)
C101—P1—C2	102.3 (3)	C306—C301—P3	121.1 (6)
C107—P1—Ir1	115.4 (2)	C301—C302—C303	120.0 (9)
C101—P1—Ir1	124.54 (19)	C301—C302—H302	120.0
C2—P1—Ir1	106.97 (18)	C303—C302—H302	120.0
C1—P2—C207	111.2 (3)	C304—C303—C302	118.5 (10)
C1—P2—C2	103.4 (2)	C304—C303—H303	120.8
C207—P2—C2	112.2 (3)	C302—C303—H303	120.8
C1—P2—C201	115.6 (3)	C305—C304—C303	121.9 (9)
C207—P2—C201	106.5 (3)	C305—C304—H304	119.0
C2—P2—C201	108.0 (3)	C303—C304—H304	119.0
C301—P3—C307	101.4 (3)	C304—C305—C306	121.2 (9)
C301—P3—C3	108.6 (3)	C304—C305—H305	119.4
C307—P3—C3	103.9 (3)	C306—C305—H305	119.4
C301—P3—Ir1	122.3 (2)	C305—C306—C301	118.4 (9)
C307—P3—Ir1	124.5 (2)	C305—C306—H306	120.8
C3—P3—Ir1	93.54 (19)	C301—C306—H306	120.8
C301—P3—P4	138.8 (2)	C312—C307—C308	118.3 (6)
C307—P3—P4	113.3 (2)	C312—C307—P3	120.9 (5)
C3—P3—P4	43.06 (19)	C308—C307—P3	120.7 (5)
Ir1—P3—P4	52.76 (4)	C309—C308—C307	120.9 (8)
C401—P4—C407	101.7 (3)	C309—C308—H308	119.5
C401—P4—C3	109.2 (3)	C307—C308—H308	119.5
C407—P4—C3	107.9 (3)	C308—C309—C310	119.3 (8)
C401—P4—Ir1	126.1 (2)	C308—C309—H309	120.4
C407—P4—Ir1	113.5 (2)	C310—C309—H309	120.4
C3—P4—Ir1	97.6 (2)	C311—C310—C309	120.6 (8)
C401—P4—P3	143.7 (2)	C311—C310—H310	119.7
C407—P4—P3	109.19 (19)	C309—C310—H310	119.7
C3—P4—P3	43.4 (2)	C310—C311—C312	119.6 (9)
Ir1—P4—P3	56.54 (5)	C310—C311—H311	120.2
N2—N1—C4	111.5 (4)	C312—C311—H311	120.2
N2—N1—Ir1	119.6 (3)	C307—C312—C311	121.1 (8)
C4—N1—Ir1	128.9 (4)	C307—C312—H312	119.4
N3—N2—N1	112.3 (4)	C311—C312—H312	119.4
N2—N3—C1	115.2 (4)	C406—C401—C402	119.2 (6)
N3—C1—P2	115.9 (4)	C406—C401—P4	123.4 (5)
N3—C1—Ir1	118.5 (4)	C402—C401—P4	117.4 (5)
P2—C1—Ir1	124.5 (3)	C403—C402—C401	120.3 (7)
P2—C2—P1	109.6 (3)	C403—C402—H402	119.8
P2—C2—H2A	109.7	C401—C402—H402	119.8
P1—C2—H2A	109.7	C404—C403—C402	119.8 (8)
P2—C2—H2B	109.7	C404—C403—H403	120.1
P1—C2—H2B	109.7	C402—C403—H403	120.1
H2A—C2—H2B	108.2	C403—C404—C405	121.2 (8)
P4—C3—P3	93.5 (3)	C403—C404—H404	119.4
P4—C3—H3A	113.0	C405—C404—H404	119.4
P3—C3—H3A	113.0	C404—C405—C406	120.4 (8)
P4—C3—H3B	113.0	C404—C405—H405	119.8
P3—C3—H3B	113.0	C406—C405—H405	119.8
H3A—C3—H3B	110.4	C401—C406—C405	119.0 (8)
C5—C4—C9	119.7 (5)	C401—C406—H406	120.5
C5—C4—N1	119.2 (5)	C405—C406—H406	120.5
C9—C4—N1	121.1 (5)	C408—C407—C412	119.2 (6)
C4—C5—C6	120.5 (6)	C408—C407—P4	120.2 (4)
C4—C5—H5	119.7	C412—C407—P4	120.6 (5)
C6—C5—H5	119.7	C407—C408—C409	119.9 (6)
C5—C6—C7	118.4 (6)	C407—C408—H408	120.1
C5—C6—H6	120.8	C409—C408—H408	120.1
C7—C6—H6	120.8	C410—C409—C408	119.4 (7)
C8—C7—C6	122.0 (6)	C410—C409—H409	120.3
C8—C7—Cl2	119.0 (6)	C408—C409—H409	120.3
C6—C7—Cl2	118.9 (6)	C409—C410—C411	121.4 (7)
C7—C8—C9	118.9 (6)	C409—C410—H410	119.3
C7—C8—H8	120.5	C411—C410—H410	119.3
C9—C8—H8	120.5	C410—C411—C412	119.6 (7)
C4—C9—C8	120.4 (6)	C410—C411—H411	120.2
C4—C9—H9	119.8	C412—C411—H411	120.2
C8—C9—H9	119.8	C411—C412—C407	120.4 (7)
C106—C101—C102	118.6 (6)	C411—C412—H412	119.8
C106—C101—P1	123.3 (5)	C407—C412—H412	119.8
C102—C101—P1	117.9 (5)	C17—C12—C13	116.2 (15)
C101—C102—C103	119.2 (8)	C17—C12—C18	122.0 (18)
C101—C102—H102	120.4	C13—C12—C18	119.9 (18)
C103—C102—H102	120.4	C14—C13—C12	120.4 (15)
C104—C103—C102	119.7 (7)	C14—C13—H13	119.8
C104—C103—H103	120.1	C12—C13—H13	119.8
C102—C103—H103	120.1	C13—C14—C15	120.2 (16)
C105—C104—C103	121.4 (8)	C13—C14—H14	119.9
C105—C104—H104	119.3	C15—C14—H14	119.9
C103—C104—H104	119.3	C14—C15—C16	118.2 (16)
C104—C105—C106	119.2 (9)	C14—C15—H15	120.9
C104—C105—H105	120.4	C16—C15—H15	120.9
C106—C105—H105	120.4	C17—C16—C15	120.0 (15)
C101—C106—C105	121.8 (7)	C17—C16—H16	120.0
C101—C106—H106	119.1	C15—C16—H16	120.0
C105—C106—H106	119.1	C16—C17—C12	121.7 (15)
C108—C107—C112	118.0 (6)	C16—C17—H17	119.1
C108—C107—P1	120.8 (5)	C12—C17—H17	119.1
C112—C107—P1	121.1 (6)	C12—C18—H18A	109.5
C107—C108—C109	120.7 (8)	C12—C18—H18B	109.5
C107—C108—H108	119.7	H18A—C18—H18B	109.5
C109—C108—H108	119.7	C12—C18—H18C	109.5
C110—C109—C108	120.3 (10)	H18A—C18—H18C	109.5
C110—C109—H109	119.8	H18B—C18—H18C	109.5
C108—C109—H109	119.8	C24—C19—C20	115.5 (14)
C109—C110—C111	120.2 (8)	C24—C19—C25	121.4 (16)
C109—C110—H110	119.9	C20—C19—C25	122.5 (16)
C111—C110—H110	119.9	C21—C20—C19	120.4 (15)
C112—C111—C110	119.8 (9)	C21—C20—H20	119.8
C112—C111—H111	120.1	C19—C20—H20	119.8
C110—C111—H111	120.1	C22—C21—C20	120.5 (15)
C111—C112—C107	121.1 (9)	C22—C21—H21	119.7
C111—C112—H112	119.5	C20—C21—H21	119.7
C107—C112—H112	119.5	C21—C22—C23	119.9 (15)
C202—C201—C206	119.9 (6)	C21—C22—H22	120.0
C202—C201—P2	121.9 (5)	C23—C22—H22	120.0
C206—C201—P2	118.3 (5)	C24—C23—C22	119.6 (15)
C201—C202—C203	120.1 (7)	C24—C23—H23	120.2
C201—C202—H202	120.0	C22—C23—H23	120.2
C203—C202—H202	120.0	C23—C24—C19	120.9 (17)
C204—C203—C202	120.3 (7)	C23—C24—H24	119.5
C204—C203—H203	119.8	C19—C24—H24	119.5
C202—C203—H203	119.8	C19—C25—H25A	109.5
C203—C204—C205	119.9 (7)	C19—C25—H25B	109.5
C203—C204—H204	120.0	H25A—C25—H25B	109.5
C205—C204—H204	120.0	C19—C25—H25C	109.5
C206—C205—C204	120.5 (7)	H25A—C25—H25C	109.5
C206—C205—H205	119.7	H25B—C25—H25C	109.5
C204—C205—H205	119.7	Cl4—C10—Cl3	116.1 (11)
C205—C206—C201	119.2 (7)	Cl4—C10—H10A	108.3
C205—C206—H206	120.4	Cl3—C10—H10A	108.3
C201—C206—H206	120.4	Cl4—C10—H10B	108.3
C208—C207—C212	119.9 (6)	Cl3—C10—H10B	108.3
C208—C207—P2	117.1 (5)	H10A—C10—H10B	107.4
C212—C207—P2	123.0 (5)	Cl5—C11—Cl6	115.1 (8)
C207—C208—C209	119.8 (7)	Cl5—C11—H11A	108.5
C207—C208—H208	120.1	Cl6—C11—H11A	108.5
C209—C208—H208	120.1	Cl5—C11—H11B	108.5
C210—C209—C208	121.6 (8)	Cl6—C11—H11B	108.5
C210—C209—H209	119.2	H11A—C11—H11B	107.5
			
C4—N1—N2—N3	−173.9 (4)	C207—C208—C209—C210	2.8 (12)
Ir1—N1—N2—N3	8.1 (6)	C208—C209—C210—C211	−3.8 (13)
N1—N2—N3—C1	−3.1 (7)	C209—C210—C211—C212	1.5 (13)
N2—N3—C1—P2	165.7 (4)	C208—C207—C212—C211	−2.7 (11)
N2—N3—C1—Ir1	−3.0 (6)	P2—C207—C212—C211	175.1 (6)
C207—P2—C1—N3	−47.9 (5)	C210—C211—C212—C207	1.7 (12)
C2—P2—C1—N3	−168.5 (4)	C307—P3—C301—C302	105.5 (6)
C201—P2—C1—N3	73.7 (5)	C3—P3—C301—C302	−145.5 (6)
C207—P2—C1—Ir1	120.0 (4)	Ir1—P3—C301—C302	−38.6 (7)
C2—P2—C1—Ir1	−0.6 (4)	P4—P3—C301—C302	−107.2 (6)
C201—P2—C1—Ir1	−118.4 (3)	C307—P3—C301—C306	−72.4 (6)
C1—P2—C2—P1	22.3 (4)	C3—P3—C301—C306	36.6 (6)
C207—P2—C2—P1	−97.7 (3)	Ir1—P3—C301—C306	143.5 (5)
C201—P2—C2—P1	145.3 (3)	P4—P3—C301—C306	74.9 (7)
C107—P1—C2—P2	88.6 (3)	C306—C301—C302—C303	−0.9 (12)
C101—P1—C2—P2	−165.8 (3)	P3—C301—C302—C303	−178.8 (7)
Ir1—P1—C2—P2	−33.4 (3)	C301—C302—C303—C304	0.7 (15)
C401—P4—C3—P3	−150.9 (3)	C302—C303—C304—C305	0.3 (17)
C407—P4—C3—P3	99.3 (3)	C303—C304—C305—C306	−1.1 (17)
Ir1—P4—C3—P3	−18.4 (3)	C304—C305—C306—C301	0.8 (14)
C301—P3—C3—P4	143.3 (3)	C302—C301—C306—C305	0.1 (11)
C307—P3—C3—P4	−109.4 (3)	P3—C301—C306—C305	178.1 (6)
Ir1—P3—C3—P4	17.4 (2)	C301—P3—C307—C312	150.3 (7)
N2—N1—C4—C5	110.7 (6)	C3—P3—C307—C312	37.6 (7)
Ir1—N1—C4—C5	−71.5 (7)	Ir1—P3—C307—C312	−66.7 (7)
N2—N1—C4—C9	−69.1 (7)	P4—P3—C307—C312	−6.9 (7)
Ir1—N1—C4—C9	108.6 (6)	C301—P3—C307—C308	−28.8 (6)
C9—C4—C5—C6	−0.7 (10)	C3—P3—C307—C308	−141.4 (6)
N1—C4—C5—C6	179.4 (6)	Ir1—P3—C307—C308	114.3 (5)
C4—C5—C6—C7	−0.7 (10)	P4—P3—C307—C308	174.1 (5)
C5—C6—C7—C8	2.0 (11)	C312—C307—C308—C309	−1.7 (11)
C5—C6—C7—Cl2	−175.9 (5)	P3—C307—C308—C309	177.4 (6)
C6—C7—C8—C9	−1.9 (10)	C307—C308—C309—C310	2.0 (13)
Cl2—C7—C8—C9	176.0 (5)	C308—C309—C310—C311	−1.6 (15)
C5—C4—C9—C8	0.8 (9)	C309—C310—C311—C312	1.0 (16)
N1—C4—C9—C8	−179.3 (5)	C308—C307—C312—C311	1.0 (13)
C7—C8—C9—C4	0.5 (10)	P3—C307—C312—C311	−178.0 (8)
C107—P1—C101—C106	128.3 (6)	C310—C311—C312—C307	−0.7 (16)
C2—P1—C101—C106	22.1 (6)	C407—P4—C401—C406	106.9 (6)
Ir1—P1—C101—C106	−98.8 (6)	C3—P4—C401—C406	−7.0 (7)
C107—P1—C101—C102	−55.4 (5)	Ir1—P4—C401—C406	−122.2 (6)
C2—P1—C101—C102	−161.7 (5)	P3—P4—C401—C406	−41.4 (8)
Ir1—P1—C101—C102	77.5 (5)	C407—P4—C401—C402	−71.2 (5)
C106—C101—C102—C103	2.0 (10)	C3—P4—C401—C402	175.0 (5)
P1—C101—C102—C103	−174.4 (6)	Ir1—P4—C401—C402	59.8 (6)
C101—C102—C103—C104	−1.1 (12)	P3—P4—C401—C402	140.6 (4)
C102—C103—C104—C105	−1.0 (14)	C406—C401—C402—C403	0.9 (10)
C103—C104—C105—C106	2.2 (14)	P4—C401—C402—C403	179.0 (6)
C102—C101—C106—C105	−0.8 (11)	C401—C402—C403—C404	0.7 (12)
P1—C101—C106—C105	175.4 (6)	C402—C403—C404—C405	−1.7 (14)
C104—C105—C106—C101	−1.3 (13)	C403—C404—C405—C406	1.0 (15)
C101—P1—C107—C108	145.3 (6)	C402—C401—C406—C405	−1.6 (11)
C2—P1—C107—C108	−108.9 (6)	P4—C401—C406—C405	−179.6 (6)
Ir1—P1—C107—C108	7.2 (6)	C404—C405—C406—C401	0.7 (13)
C101—P1—C107—C112	−39.5 (6)	C401—P4—C407—C408	123.5 (5)
C2—P1—C107—C112	66.3 (6)	C3—P4—C407—C408	−121.7 (5)
Ir1—P1—C107—C112	−177.6 (5)	Ir1—P4—C407—C408	−14.8 (6)
C112—C107—C108—C109	0.8 (11)	P3—P4—C407—C408	−75.7 (5)
P1—C107—C108—C109	176.1 (7)	C401—P4—C407—C412	−58.2 (6)
C107—C108—C109—C110	−0.8 (15)	C3—P4—C407—C412	56.7 (6)
C108—C109—C110—C111	−0.2 (17)	Ir1—P4—C407—C412	163.6 (4)
C109—C110—C111—C112	1.2 (16)	P3—P4—C407—C412	102.6 (5)
C110—C111—C112—C107	−1.2 (13)	C412—C407—C408—C409	−2.7 (10)
C108—C107—C112—C111	0.2 (11)	P4—C407—C408—C409	175.7 (5)
P1—C107—C112—C111	−175.1 (6)	C407—C408—C409—C410	0.5 (11)
C1—P2—C201—C202	−14.3 (6)	C408—C409—C410—C411	2.0 (12)
C207—P2—C201—C202	109.8 (6)	C409—C410—C411—C412	−2.3 (12)
C2—P2—C201—C202	−129.6 (5)	C410—C411—C412—C407	0.1 (11)
C1—P2—C201—C206	164.5 (5)	C408—C407—C412—C411	2.4 (10)
C207—P2—C201—C206	−71.4 (6)	P4—C407—C412—C411	−175.9 (5)
C2—P2—C201—C206	49.2 (6)	C17—C12—C13—C14	−11 (10)
C206—C201—C202—C203	1.1 (10)	C18—C12—C13—C14	−175 (7)
P2—C201—C202—C203	179.8 (6)	C12—C13—C14—C15	−4 (10)
C201—C202—C203—C204	−0.5 (12)	C13—C14—C15—C16	17 (9)
C202—C203—C204—C205	−0.5 (13)	C14—C15—C16—C17	−16 (8)
C203—C204—C205—C206	0.8 (13)	C15—C16—C17—C12	1 (12)
C204—C205—C206—C201	−0.2 (12)	C13—C12—C17—C16	13 (12)
C202—C201—C206—C205	−0.8 (10)	C18—C12—C17—C16	177 (8)
P2—C201—C206—C205	−179.6 (6)	C24—C19—C20—C21	14 (9)
C1—P2—C207—C208	74.4 (6)	C25—C19—C20—C21	−175 (6)
C2—P2—C207—C208	−170.3 (5)	C19—C20—C21—C22	1 (9)
C201—P2—C207—C208	−52.4 (6)	C20—C21—C22—C23	−11 (8)
C1—P2—C207—C212	−103.4 (6)	C21—C22—C23—C24	4 (9)
C2—P2—C207—C212	11.9 (7)	C22—C23—C24—C19	12 (9)
C201—P2—C207—C212	129.8 (6)	C20—C19—C24—C23	−20 (9)
C212—C207—C208—C209	0.5 (11)	C25—C19—C24—C23	168 (6)
P2—C207—C208—C209	−177.4 (6)		

### [7-(4-Chlorophenyl)-1,1,3,3-tetraphenyl-5,6,7-triaza-κN7-1,3λ4-diphospha-κP1-hepta-4,6-dien-4-yl][methylenebis(diphenylphosphine)-κ2P,P']iridium(I) chloride–dichloromethane–toluene (2/3/1) (2) Hydrogen-bond geometry (Å, º)

**Table:**

*D*—H···*A*	*D*—H	H···*A*	*D*···*A*	*D*—H···*A*
C2—H2*B*···Cl1	0.98	2.62	3.5215 (1)	153
C11—H11*B*···Cl1^i^	0.98	2.49	3.4594 (1)	170
C209—H209···N3^ii^	0.94	2.60	3.3722 (1)	140
C306—H306···Cl1*A*^iii^	0.94	2.81	3.7037 (1)	159

Symmetry codes: (i) −*x*+1/2, *y*+1/2, *z*; (ii) −*x*+1, *y*, −*z*−1/2; (iii) −*x*+1/2, −*y*+3/2, *z*+1/2.

### {4-[3-(4-Chlorophenyl)triazenido-κN3]-1,1,3,3-tetraphenyl-1,3λ5-diphospha-κP1-but-2-en-4-yl}cyanido[methylenebis(diphenylphosphine)-κ2P,P']iridium(III) methanol disolvate (3) Crystal data

**Table:**

[Ir(CN)(C_23_H_22_P_2_)(C_34_H_26_ClN_3_P_2_)]·2CH_4_O	*Z* = 2
*M**_r_* = 1216.61	*F*(000) = 1228
Triclinic, *P*1	*D*_x_ = 1.521 Mg m^−^^3^
*a* = 11.1683 (1) Å	Mo *K*α radiation, λ = 0.71073 Å
*b* = 12.7805 (2) Å	Cell parameters from 31262 reflections
*c* = 20.0591 (3) Å	θ = 1.0–27.5°
α = 98.475 (1)°	µ = 2.73 mm^−^^1^
β = 93.122 (1)°	*T* = 223 K
γ = 109.336 (1)°	Prism, colourless
*V* = 2655.75 (6) Å^3^	0.31 × 0.30 × 0.12 mm

### {4-[3-(4-Chlorophenyl)triazenido-κN3]-1,1,3,3-tetraphenyl-1,3λ5-diphospha-κP1-but-2-en-4-yl}cyanido[methylenebis(diphenylphosphine)-κ2P,P']iridium(III) methanol disolvate (3) Data collection

**Table:**

Nonius KappaCCD diffractometer	*R*_int_ = 0.034
phi– and ω–scans	θ_max_ = 26.0°, θ_min_ = 1.7°
20072 measured reflections	*h* = −13→13
10396 independent reflections	*k* = −15→15
9895 reflections with *I* > 2σ(*I*)	*l* = −24→24

### {4-[3-(4-Chlorophenyl)triazenido-κN3]-1,1,3,3-tetraphenyl-1,3λ5-diphospha-κP1-but-2-en-4-yl}cyanido[methylenebis(diphenylphosphine)-κ2P,P']iridium(III) methanol disolvate (3) Refinement

**Table:**

Refinement on *F*^2^	0 restraints
Least-squares matrix: full	Hydrogen site location: mixed
*R*[*F*^2^ > 2σ(*F*^2^)] = 0.023	H atoms treated by a mixture of independent and constrained refinement
*wR*(*F*^2^) = 0.056	*w* = 1/[σ^2^(*F*_o_^2^) + (0.0184*P*)^2^ + 2.0572*P*] where *P* = (*F*_o_^2^ + 2*F*_c_^2^)/3
*S* = 1.05	(Δ/σ)_max_ = 0.002
10396 reflections	Δρ_max_ = 0.77 e Å^−^^3^
661 parameters	Δρ_min_ = −1.10 e Å^−^^3^

### {4-[3-(4-Chlorophenyl)triazenido-κN3]-1,1,3,3-tetraphenyl-1,3λ5-diphospha-κP1-but-2-en-4-yl}cyanido[methylenebis(diphenylphosphine)-κ2P,P']iridium(III) methanol disolvate (3) Special details

**Table:**

Experimental. All data sets were measured with several scans to increase the number of redundant reflections. In our experience this method of averaging redundant reflections replaces in a good approximation semi-empirical absorption methods (absorption correction programs like SORTAV lead to no better data sets).
Geometry. All esds (except the esd in the dihedral angle between two l.s. planes) are estimated using the full covariance matrix. The cell esds are taken into account individually in the estimation of esds in distances, angles and torsion angles; correlations between esds in cell parameters are only used when they are defined by crystal symmetry. An approximate (isotropic) treatment of cell esds is used for estimating esds involving l.s. planes.
Refinement. Hydrogen atoms at C1 and C2 were localized and refined with isotropic displacement parameters.

### {4-[3-(4-Chlorophenyl)triazenido-κN3]-1,1,3,3-tetraphenyl-1,3λ5-diphospha-κP1-but-2-en-4-yl}cyanido[methylenebis(diphenylphosphine)-κ2P,P']iridium(III) methanol disolvate (3) Fractional atomic coordinates and isotropic or equivalent isotropic displacement parameters (Å^2^)

**Table:**

	*x*	*y*	*z*	*U*_iso_*/*U*_eq_	
Ir1	0.35118 (2)	0.22849 (2)	0.74840 (2)	0.01977 (4)	
Cl1	−0.33530 (7)	−0.10915 (7)	0.67497 (5)	0.0557 (2)	
P1	0.37841 (6)	0.34073 (5)	0.85641 (3)	0.02148 (13)	
P2	0.46189 (6)	0.51310 (5)	0.77072 (3)	0.02315 (13)	
P3	0.36652 (6)	0.12853 (6)	0.64246 (3)	0.02637 (14)	
P4	0.54330 (6)	0.19402 (5)	0.75590 (3)	0.02464 (14)	
N1	0.17849 (19)	0.24596 (18)	0.71335 (11)	0.0256 (5)	
N2	0.1886 (2)	0.31929 (18)	0.67036 (11)	0.0294 (5)	
N3	0.2975 (2)	0.38693 (18)	0.66488 (11)	0.0298 (5)	
C1	0.4080 (2)	0.3808 (2)	0.70859 (13)	0.0238 (5)	
H1	0.473 (3)	0.388 (3)	0.6813 (16)	0.040 (8)*	
C2	0.4452 (2)	0.4807 (2)	0.84909 (14)	0.0280 (6)	
H2	0.455 (3)	0.539 (2)	0.8856 (15)	0.033 (8)*	
C3	0.5057 (3)	0.0974 (2)	0.67372 (14)	0.0319 (6)	
H3A	0.5750	0.1169	0.6448	0.038*	
H3B	0.4840	0.0185	0.6791	0.038*	
C4	0.0537 (2)	0.1642 (2)	0.70456 (14)	0.0273 (5)	
C5	0.0021 (2)	0.1141 (2)	0.75831 (14)	0.0310 (6)	
H5	0.0487	0.1373	0.8015	0.037*	
C6	−0.1164 (3)	0.0310 (2)	0.74920 (16)	0.0359 (6)	
H6	−0.1502	−0.0022	0.7860	0.043*	
C7	−0.1853 (3)	−0.0034 (2)	0.68569 (17)	0.0384 (7)	
C8	−0.1381 (3)	0.0446 (3)	0.63178 (16)	0.0403 (7)	
H8	−0.1864	0.0212	0.5890	0.048*	
C9	−0.0181 (3)	0.1284 (2)	0.64053 (15)	0.0359 (6)	
H9	0.0149	0.1611	0.6034	0.043*	
C101	0.2366 (2)	0.3316 (2)	0.90152 (13)	0.0260 (5)	
C102	0.1406 (2)	0.3617 (2)	0.87082 (14)	0.0319 (6)	
H102	0.1458	0.3780	0.8267	0.038*	
C103	0.0374 (3)	0.3679 (3)	0.90492 (16)	0.0404 (7)	
H103	−0.0265	0.3886	0.8838	0.048*	
C104	0.0282 (3)	0.3438 (3)	0.96972 (18)	0.0487 (8)	
H104	−0.0413	0.3486	0.9928	0.058*	
C105	0.1213 (3)	0.3129 (3)	1.00026 (17)	0.0511 (8)	
H105	0.1146	0.2956	1.0442	0.061*	
C106	0.2251 (3)	0.3070 (3)	0.96686 (15)	0.0381 (7)	
H106	0.2883	0.2861	0.9885	0.046*	
C107	0.4795 (2)	0.3081 (2)	0.91953 (13)	0.0261 (5)	
C108	0.5897 (3)	0.3915 (2)	0.95340 (14)	0.0333 (6)	
H108	0.6125	0.4648	0.9435	0.040*	
C109	0.6661 (3)	0.3677 (3)	1.00168 (15)	0.0425 (7)	
H109	0.7408	0.4245	1.0238	0.051*	
C110	0.6325 (3)	0.2609 (3)	1.01710 (16)	0.0462 (8)	
H110	0.6839	0.2447	1.0499	0.055*	
C111	0.5228 (3)	0.1775 (3)	0.98416 (15)	0.0405 (7)	
H111	0.4995	0.1048	0.9950	0.049*	
C112	0.4471 (3)	0.2003 (2)	0.93538 (14)	0.0321 (6)	
H112	0.3734	0.1427	0.9128	0.038*	
C201	0.3767 (2)	0.6072 (2)	0.75281 (14)	0.0266 (5)	
C202	0.2929 (3)	0.6294 (2)	0.79717 (16)	0.0365 (6)	
H202	0.2773	0.5932	0.8350	0.044*	
C203	0.2323 (3)	0.7053 (3)	0.78555 (19)	0.0472 (8)	
H203	0.1770	0.7213	0.8160	0.057*	
C204	0.2527 (3)	0.7568 (3)	0.7300 (2)	0.0506 (9)	
H204	0.2104	0.8071	0.7222	0.061*	
C205	0.3354 (3)	0.7352 (3)	0.68530 (19)	0.0482 (8)	
H205	0.3487	0.7703	0.6470	0.058*	
C206	0.3984 (3)	0.6617 (2)	0.69712 (16)	0.0370 (7)	
H206	0.4561	0.6485	0.6674	0.044*	
C207	0.6230 (2)	0.5994 (2)	0.75804 (15)	0.0315 (6)	
C208	0.6933 (3)	0.6839 (2)	0.81107 (18)	0.0424 (7)	
H208	0.6584	0.6933	0.8522	0.051*	
C209	0.8150 (3)	0.7547 (3)	0.8039 (2)	0.0545 (10)	
H209	0.8617	0.8120	0.8401	0.065*	
C210	0.8674 (3)	0.7416 (3)	0.7443 (2)	0.0600 (11)	
H210	0.9498	0.7896	0.7397	0.072*	
C211	0.7991 (3)	0.6580 (3)	0.6913 (2)	0.0559 (9)	
H211	0.8356	0.6485	0.6507	0.067*	
C212	0.6765 (3)	0.5873 (3)	0.69726 (17)	0.0418 (7)	
H212	0.6296	0.5315	0.6604	0.050*	
C301	0.2520 (3)	−0.0063 (2)	0.59993 (14)	0.0329 (6)	
C302	0.1351 (3)	−0.0519 (2)	0.62255 (16)	0.0400 (7)	
H302	0.1138	−0.0139	0.6613	0.048*	
C303	0.0481 (4)	−0.1541 (3)	0.58850 (19)	0.0555 (9)	
H303	−0.0323	−0.1836	0.6038	0.067*	
C304	0.0787 (4)	−0.2118 (3)	0.53297 (19)	0.0611 (10)	
H304	0.0193	−0.2802	0.5098	0.073*	
C305	0.1971 (4)	−0.1687 (3)	0.51126 (19)	0.0647 (11)	
H305	0.2195	−0.2094	0.4740	0.078*	
C306	0.2839 (4)	−0.0658 (3)	0.54393 (17)	0.0510 (8)	
H306	0.3640	−0.0364	0.5282	0.061*	
C307	0.4113 (3)	0.2083 (2)	0.57471 (14)	0.0356 (6)	
C308	0.3161 (4)	0.2147 (3)	0.52929 (16)	0.0523 (9)	
H308	0.2305	0.1703	0.5305	0.063*	
C309	0.3469 (5)	0.2860 (4)	0.48258 (19)	0.0697 (12)	
H309	0.2820	0.2906	0.4525	0.084*	
C310	0.4706 (6)	0.3496 (4)	0.4798 (2)	0.0771 (14)	
H310	0.4903	0.3979	0.4477	0.092*	
C311	0.5669 (5)	0.3443 (4)	0.5230 (2)	0.0754 (13)	
H311	0.6522	0.3879	0.5204	0.091*	
C312	0.5367 (4)	0.2732 (3)	0.57100 (19)	0.0544 (9)	
H312	0.6022	0.2694	0.6010	0.065*	
C401	0.5894 (2)	0.1128 (2)	0.81428 (13)	0.0273 (5)	
C402	0.5127 (3)	0.0026 (2)	0.81453 (15)	0.0346 (6)	
H402	0.4341	−0.0282	0.7871	0.041*	
C403	0.5503 (3)	−0.0625 (3)	0.85466 (17)	0.0419 (7)	
H403	0.4980	−0.1374	0.8541	0.050*	
C404	0.6653 (3)	−0.0170 (3)	0.89567 (17)	0.0447 (8)	
H404	0.6916	−0.0612	0.9228	0.054*	
C405	0.7413 (3)	0.0928 (3)	0.89676 (16)	0.0426 (7)	
H405	0.8186	0.1237	0.9253	0.051*	
C406	0.7048 (2)	0.1581 (2)	0.85609 (15)	0.0335 (6)	
H406	0.7577	0.2329	0.8567	0.040*	
C407	0.6942 (2)	0.3041 (2)	0.75248 (15)	0.0314 (6)	
C408	0.7255 (2)	0.4061 (2)	0.79592 (15)	0.0335 (6)	
H408	0.6652	0.4208	0.8233	0.040*	
C409	0.8465 (3)	0.4872 (3)	0.79924 (19)	0.0458 (8)	
H409	0.8689	0.5557	0.8297	0.055*	
C410	0.9334 (3)	0.4660 (3)	0.7573 (2)	0.0532 (9)	
H410	1.0137	0.5217	0.7581	0.064*	
C411	0.9036 (3)	0.3646 (3)	0.7146 (2)	0.0526 (9)	
H411	0.9636	0.3506	0.6867	0.063*	
C412	0.7852 (3)	0.2833 (3)	0.71273 (18)	0.0429 (7)	
H412	0.7657	0.2131	0.6843	0.051*	
N4	0.2110 (2)	0.0011 (2)	0.80197 (13)	0.0404 (6)	
C10	0.2640 (2)	0.0841 (2)	0.78323 (13)	0.0263 (5)	
O2	0.1944 (4)	0.4619 (3)	0.54949 (16)	0.0904 (10)	
H2A	0.2429	0.4526	0.5791	0.136*	
C12	0.1072 (5)	0.5034 (4)	0.5799 (3)	0.0897 (16)	
H12A	0.1330	0.5260	0.6283	0.135*	
H12B	0.0234	0.4453	0.5719	0.135*	
H12C	0.1041	0.5680	0.5608	0.135*	
O1	0.1200 (6)	−0.1236 (4)	0.9047 (3)	0.1429 (19)	
H1A	0.1484	−0.0999	0.8703	0.214*	
C11	0.0930 (7)	−0.0440 (5)	0.9440 (3)	0.120 (2)	
H11A	0.0488	−0.0747	0.9808	0.180*	
H11B	0.0390	−0.0157	0.9176	0.180*	
H11C	0.1716	0.0170	0.9624	0.180*	

### {4-[3-(4-Chlorophenyl)triazenido-κN3]-1,1,3,3-tetraphenyl-1,3λ5-diphospha-κP1-but-2-en-4-yl}cyanido[methylenebis(diphenylphosphine)-κ2P,P']iridium(III) methanol disolvate (3) Atomic displacement parameters (Å^2^)

**Table:**

	*U*^11^	*U*^22^	*U*^33^	*U*^12^	*U*^13^	*U*^23^
Ir1	0.02111 (5)	0.01730 (6)	0.02081 (6)	0.00605 (4)	0.00309 (4)	0.00405 (4)
Cl1	0.0292 (4)	0.0364 (4)	0.0900 (7)	0.0023 (3)	−0.0012 (4)	−0.0010 (4)
P1	0.0230 (3)	0.0193 (3)	0.0215 (3)	0.0062 (2)	0.0027 (2)	0.0037 (2)
P2	0.0222 (3)	0.0183 (3)	0.0288 (3)	0.0060 (2)	0.0034 (2)	0.0059 (3)
P3	0.0339 (3)	0.0246 (3)	0.0225 (3)	0.0125 (3)	0.0043 (3)	0.0038 (3)
P4	0.0233 (3)	0.0224 (3)	0.0292 (4)	0.0087 (3)	0.0040 (3)	0.0053 (3)
N1	0.0226 (10)	0.0280 (11)	0.0267 (11)	0.0092 (9)	−0.0002 (8)	0.0058 (9)
N2	0.0331 (12)	0.0272 (12)	0.0285 (12)	0.0120 (10)	−0.0019 (9)	0.0049 (9)
N3	0.0378 (12)	0.0251 (12)	0.0262 (12)	0.0105 (10)	0.0011 (9)	0.0058 (9)
C1	0.0250 (12)	0.0229 (13)	0.0234 (13)	0.0065 (10)	0.0068 (10)	0.0063 (10)
C2	0.0341 (14)	0.0190 (13)	0.0286 (14)	0.0061 (10)	0.0046 (11)	0.0043 (11)
C3	0.0355 (14)	0.0324 (15)	0.0326 (15)	0.0187 (12)	0.0060 (11)	0.0027 (12)
C4	0.0216 (12)	0.0269 (14)	0.0337 (14)	0.0099 (10)	0.0014 (10)	0.0028 (11)
C5	0.0321 (13)	0.0269 (14)	0.0324 (15)	0.0098 (11)	0.0024 (11)	0.0017 (11)
C6	0.0317 (14)	0.0300 (15)	0.0445 (17)	0.0094 (12)	0.0092 (12)	0.0031 (13)
C7	0.0252 (13)	0.0257 (14)	0.058 (2)	0.0056 (11)	−0.0009 (13)	−0.0025 (13)
C8	0.0339 (15)	0.0384 (17)	0.0422 (18)	0.0112 (13)	−0.0099 (13)	−0.0044 (14)
C9	0.0319 (14)	0.0399 (16)	0.0306 (15)	0.0079 (12)	−0.0014 (11)	0.0026 (12)
C101	0.0264 (12)	0.0198 (12)	0.0292 (14)	0.0051 (10)	0.0062 (10)	0.0016 (10)
C102	0.0285 (13)	0.0315 (15)	0.0317 (15)	0.0073 (11)	0.0005 (11)	0.0016 (12)
C103	0.0251 (13)	0.0425 (17)	0.0504 (19)	0.0096 (12)	0.0041 (12)	0.0036 (14)
C104	0.0331 (16)	0.057 (2)	0.055 (2)	0.0131 (14)	0.0196 (14)	0.0068 (17)
C105	0.055 (2)	0.066 (2)	0.0386 (18)	0.0221 (17)	0.0223 (15)	0.0181 (17)
C106	0.0401 (15)	0.0479 (18)	0.0336 (16)	0.0201 (14)	0.0127 (12)	0.0150 (13)
C107	0.0291 (12)	0.0287 (14)	0.0215 (12)	0.0121 (11)	0.0015 (10)	0.0028 (10)
C108	0.0345 (14)	0.0343 (15)	0.0286 (14)	0.0110 (12)	−0.0006 (11)	0.0015 (12)
C109	0.0392 (16)	0.0499 (19)	0.0328 (16)	0.0152 (14)	−0.0096 (13)	−0.0051 (14)
C110	0.0558 (19)	0.060 (2)	0.0303 (16)	0.0327 (17)	−0.0058 (14)	0.0049 (15)
C111	0.0593 (19)	0.0426 (18)	0.0296 (15)	0.0277 (15)	0.0075 (14)	0.0120 (13)
C112	0.0391 (15)	0.0305 (15)	0.0287 (14)	0.0136 (12)	0.0054 (11)	0.0073 (11)
C201	0.0244 (12)	0.0161 (12)	0.0360 (15)	0.0040 (9)	−0.0020 (10)	0.0030 (10)
C202	0.0319 (14)	0.0294 (15)	0.0452 (17)	0.0098 (12)	0.0014 (12)	0.0003 (13)
C203	0.0357 (16)	0.0344 (17)	0.071 (2)	0.0174 (13)	−0.0012 (15)	−0.0034 (16)
C204	0.0395 (17)	0.0250 (15)	0.085 (3)	0.0164 (13)	−0.0196 (17)	−0.0007 (16)
C205	0.0517 (19)	0.0301 (16)	0.062 (2)	0.0135 (14)	−0.0126 (16)	0.0145 (15)
C206	0.0376 (15)	0.0305 (15)	0.0429 (17)	0.0114 (12)	−0.0004 (13)	0.0090 (13)
C207	0.0234 (12)	0.0283 (14)	0.0448 (17)	0.0075 (11)	0.0012 (11)	0.0170 (12)
C208	0.0326 (15)	0.0324 (16)	0.057 (2)	0.0040 (12)	−0.0038 (14)	0.0126 (14)
C209	0.0367 (17)	0.0373 (18)	0.078 (3)	−0.0021 (14)	−0.0114 (17)	0.0171 (17)
C210	0.0294 (16)	0.057 (2)	0.093 (3)	0.0015 (15)	0.0052 (18)	0.043 (2)
C211	0.0370 (17)	0.064 (2)	0.074 (3)	0.0126 (16)	0.0211 (17)	0.040 (2)
C212	0.0318 (15)	0.0429 (18)	0.0527 (19)	0.0091 (13)	0.0087 (13)	0.0223 (15)
C301	0.0474 (16)	0.0239 (14)	0.0274 (14)	0.0144 (12)	−0.0041 (12)	0.0019 (11)
C302	0.0468 (17)	0.0289 (15)	0.0388 (17)	0.0082 (13)	0.0025 (13)	0.0008 (13)
C303	0.061 (2)	0.0341 (18)	0.058 (2)	0.0005 (15)	−0.0018 (17)	0.0047 (16)
C304	0.088 (3)	0.0293 (17)	0.050 (2)	0.0053 (18)	−0.010 (2)	−0.0013 (16)
C305	0.104 (3)	0.042 (2)	0.042 (2)	0.029 (2)	0.000 (2)	−0.0146 (16)
C306	0.069 (2)	0.0428 (19)	0.0401 (18)	0.0213 (17)	0.0081 (16)	−0.0024 (15)
C307	0.0569 (18)	0.0309 (15)	0.0254 (14)	0.0215 (13)	0.0124 (13)	0.0065 (12)
C308	0.077 (2)	0.056 (2)	0.0317 (17)	0.0309 (19)	0.0040 (16)	0.0107 (15)
C309	0.118 (4)	0.069 (3)	0.038 (2)	0.048 (3)	0.010 (2)	0.0218 (19)
C310	0.151 (5)	0.059 (3)	0.040 (2)	0.049 (3)	0.037 (3)	0.0278 (19)
C311	0.106 (3)	0.058 (3)	0.064 (3)	0.018 (2)	0.047 (3)	0.027 (2)
C312	0.063 (2)	0.052 (2)	0.052 (2)	0.0183 (17)	0.0219 (17)	0.0175 (17)
C401	0.0283 (13)	0.0273 (14)	0.0289 (14)	0.0123 (11)	0.0049 (10)	0.0059 (11)
C402	0.0331 (14)	0.0269 (14)	0.0402 (16)	0.0068 (11)	−0.0016 (12)	0.0053 (12)
C403	0.0465 (17)	0.0283 (15)	0.0509 (19)	0.0107 (13)	0.0012 (14)	0.0136 (14)
C404	0.0505 (18)	0.0455 (19)	0.0488 (19)	0.0249 (15)	0.0039 (15)	0.0219 (15)
C405	0.0335 (15)	0.0462 (18)	0.0483 (19)	0.0136 (13)	−0.0067 (13)	0.0134 (15)
C406	0.0270 (13)	0.0297 (15)	0.0423 (16)	0.0072 (11)	0.0000 (11)	0.0089 (12)
C407	0.0239 (12)	0.0308 (14)	0.0452 (17)	0.0118 (11)	0.0074 (11)	0.0171 (12)
C408	0.0256 (13)	0.0300 (15)	0.0466 (17)	0.0103 (11)	0.0015 (12)	0.0105 (13)
C409	0.0302 (15)	0.0337 (17)	0.070 (2)	0.0068 (12)	−0.0053 (14)	0.0124 (15)
C410	0.0257 (15)	0.0431 (19)	0.094 (3)	0.0092 (13)	0.0066 (16)	0.0294 (19)
C411	0.0331 (16)	0.053 (2)	0.085 (3)	0.0204 (15)	0.0261 (16)	0.0310 (19)
C412	0.0344 (15)	0.0371 (17)	0.063 (2)	0.0162 (13)	0.0164 (14)	0.0134 (15)
N4	0.0439 (14)	0.0306 (13)	0.0466 (15)	0.0086 (11)	0.0101 (12)	0.0148 (12)
C10	0.0292 (13)	0.0269 (14)	0.0247 (13)	0.0118 (11)	0.0030 (10)	0.0052 (11)
O2	0.118 (3)	0.096 (2)	0.069 (2)	0.041 (2)	0.011 (2)	0.0424 (19)
C12	0.096 (4)	0.070 (3)	0.096 (4)	0.029 (3)	−0.034 (3)	0.009 (3)
O1	0.227 (5)	0.106 (3)	0.138 (4)	0.076 (3)	0.103 (4)	0.078 (3)
C11	0.146 (6)	0.107 (5)	0.080 (4)	0.004 (4)	0.039 (4)	0.018 (3)

### {4-[3-(4-Chlorophenyl)triazenido-κN3]-1,1,3,3-tetraphenyl-1,3λ5-diphospha-κP1-but-2-en-4-yl}cyanido[methylenebis(diphenylphosphine)-κ2P,P']iridium(III) methanol disolvate (3) Geometric parameters (Å, º)

**Table:**

Ir1—P1	2.3595 (6)	C205—H205	0.9400
Ir1—P3	2.3584 (7)	C206—H206	0.9400
Ir1—P4	2.3304 (6)	C207—C208	1.387 (4)
Ir1—N1	2.1090 (19)	C207—C212	1.397 (4)
Ir1—C1	2.127 (3)	C208—C209	1.390 (4)
Ir1—C10	2.027 (3)	C208—H208	0.9400
Cl1—C7	1.747 (3)	C209—C210	1.373 (5)
P1—C2	1.727 (3)	C209—H209	0.9400
P1—C107	1.834 (2)	C210—C211	1.374 (5)
P1—C101	1.846 (3)	C210—H210	0.9400
P2—C2	1.688 (3)	C211—C212	1.392 (4)
P2—C201	1.824 (3)	C211—H211	0.9400
P2—C207	1.827 (3)	C212—H212	0.9400
P2—C1	1.843 (3)	C301—C302	1.374 (4)
P3—C307	1.811 (3)	C301—C306	1.392 (4)
P3—C301	1.823 (3)	C302—C303	1.393 (4)
P3—C3	1.829 (3)	C302—H302	0.9400
P4—C407	1.815 (3)	C303—C304	1.366 (5)
P4—C401	1.833 (3)	C303—H303	0.9400
P4—C3	1.844 (3)	C304—C305	1.376 (6)
N1—N2	1.347 (3)	C304—H304	0.9400
N1—C4	1.421 (3)	C305—C306	1.390 (5)
N2—N3	1.259 (3)	C305—H305	0.9400
N3—C1	1.504 (3)	C306—H306	0.9400
C1—H1	0.92 (3)	C307—C312	1.384 (5)
C2—H2	0.93 (3)	C307—C308	1.393 (4)
C3—H3A	0.9800	C308—C309	1.381 (5)
C3—H3B	0.9800	C308—H308	0.9400
C4—C5	1.390 (4)	C309—C310	1.360 (7)
C4—C9	1.405 (4)	C309—H309	0.9400
C5—C6	1.378 (4)	C310—C311	1.370 (7)
C5—H5	0.9400	C310—H310	0.9400
C6—C7	1.383 (4)	C311—C312	1.399 (5)
C6—H6	0.9400	C311—H311	0.9400
C7—C8	1.366 (5)	C312—H312	0.9400
C8—C9	1.394 (4)	C401—C402	1.387 (4)
C8—H8	0.9400	C401—C406	1.394 (4)
C9—H9	0.9400	C402—C403	1.382 (4)
C101—C106	1.395 (4)	C402—H402	0.9400
C101—C102	1.395 (4)	C403—C404	1.384 (4)
C102—C103	1.390 (4)	C403—H403	0.9400
C102—H102	0.9400	C404—C405	1.374 (4)
C103—C104	1.381 (5)	C404—H404	0.9400
C103—H103	0.9400	C405—C406	1.386 (4)
C104—C105	1.373 (5)	C405—H405	0.9400
C104—H104	0.9400	C406—H406	0.9400
C105—C106	1.388 (4)	C407—C408	1.382 (4)
C105—H105	0.9400	C407—C412	1.395 (4)
C106—H106	0.9400	C408—C409	1.395 (4)
C107—C108	1.393 (4)	C408—H408	0.9400
C107—C112	1.394 (4)	C409—C410	1.386 (5)
C108—C109	1.389 (4)	C409—H409	0.9400
C108—H108	0.9400	C410—C411	1.370 (5)
C109—C110	1.378 (5)	C410—H410	0.9400
C109—H109	0.9400	C411—C412	1.379 (4)
C110—C111	1.383 (5)	C411—H411	0.9400
C110—H110	0.9400	C412—H412	0.9400
C111—C112	1.383 (4)	N4—C10	1.158 (3)
C111—H111	0.9400	O2—C12	1.383 (6)
C112—H112	0.9400	O2—H2A	0.8300
C201—C206	1.392 (4)	C12—H12A	0.9700
C201—C202	1.393 (4)	C12—H12B	0.9700
C202—C203	1.392 (4)	C12—H12C	0.9700
C202—H202	0.9400	O1—C11	1.318 (7)
C203—C204	1.367 (5)	O1—H1A	0.8300
C203—H203	0.9400	C11—H11A	0.9700
C204—C205	1.387 (5)	C11—H11B	0.9700
C204—H204	0.9400	C11—H11C	0.9700
C205—C206	1.385 (4)		
			
C10—Ir1—N1	94.15 (9)	C201—C202—H202	120.0
C10—Ir1—C1	169.38 (9)	C204—C203—C202	120.4 (3)
N1—Ir1—C1	75.31 (9)	C204—C203—H203	119.8
C10—Ir1—P4	89.88 (7)	C202—C203—H203	119.8
N1—Ir1—P4	164.45 (6)	C203—C204—C205	120.3 (3)
C1—Ir1—P4	100.63 (7)	C203—C204—H204	119.9
C10—Ir1—P3	92.00 (7)	C205—C204—H204	119.9
N1—Ir1—P3	92.64 (6)	C206—C205—C204	119.9 (3)
C1—Ir1—P3	89.80 (7)	C206—C205—H205	120.1
P4—Ir1—P3	72.19 (2)	C204—C205—H205	120.1
C10—Ir1—P1	92.84 (7)	C205—C206—C201	120.3 (3)
N1—Ir1—P1	96.69 (6)	C205—C206—H206	119.8
C1—Ir1—P1	87.23 (7)	C201—C206—H206	119.8
P4—Ir1—P1	98.11 (2)	C208—C207—C212	118.8 (3)
P3—Ir1—P1	169.15 (2)	C208—C207—P2	117.7 (2)
C2—P1—C107	109.18 (12)	C212—C207—P2	123.5 (2)
C2—P1—C101	105.42 (12)	C207—C208—C209	120.5 (3)
C107—P1—C101	100.87 (12)	C207—C208—H208	119.7
C2—P1—Ir1	108.81 (10)	C209—C208—H208	119.7
C107—P1—Ir1	113.21 (8)	C210—C209—C208	120.4 (3)
C101—P1—Ir1	118.72 (8)	C210—C209—H209	119.8
C2—P2—C201	112.23 (13)	C208—C209—H209	119.8
C2—P2—C207	115.04 (13)	C209—C210—C211	119.8 (3)
C201—P2—C207	99.28 (12)	C209—C210—H210	120.1
C2—P2—C1	108.32 (13)	C211—C210—H210	120.1
C201—P2—C1	111.83 (11)	C210—C211—C212	120.6 (4)
C207—P2—C1	110.00 (13)	C210—C211—H211	119.7
C307—P3—C301	104.16 (13)	C212—C211—H211	119.7
C307—P3—C3	107.86 (14)	C211—C212—C207	119.9 (3)
C301—P3—C3	105.55 (12)	C211—C212—H212	120.1
C307—P3—Ir1	117.31 (9)	C207—C212—H212	120.1
C301—P3—Ir1	125.75 (10)	C302—C301—C306	118.9 (3)
C3—P3—Ir1	93.86 (9)	C302—C301—P3	121.5 (2)
C407—P4—C401	102.00 (12)	C306—C301—P3	119.5 (2)
C407—P4—C3	106.82 (14)	C301—C302—C303	120.5 (3)
C401—P4—C3	103.06 (12)	C301—C302—H302	119.7
C407—P4—Ir1	121.19 (9)	C303—C302—H302	119.7
C401—P4—Ir1	125.97 (9)	C304—C303—C302	120.5 (4)
C3—P4—Ir1	94.38 (8)	C304—C303—H303	119.7
N2—N1—C4	111.0 (2)	C302—C303—H303	119.7
N2—N1—Ir1	115.61 (15)	C303—C304—C305	119.4 (3)
C4—N1—Ir1	127.86 (17)	C303—C304—H304	120.3
N3—N2—N1	118.7 (2)	C305—C304—H304	120.3
N2—N3—C1	116.7 (2)	C304—C305—C306	120.6 (3)
N3—C1—P2	104.86 (16)	C304—C305—H305	119.7
N3—C1—Ir1	109.85 (16)	C306—C305—H305	119.7
P2—C1—Ir1	116.66 (13)	C305—C306—C301	119.9 (4)
N3—C1—H1	105.7 (19)	C305—C306—H306	120.1
P2—C1—H1	104.7 (19)	C301—C306—H306	120.1
Ir1—C1—H1	114.1 (19)	C312—C307—C308	118.6 (3)
P2—C2—P1	118.42 (16)	C312—C307—P3	121.8 (2)
P2—C2—H2	118.6 (18)	C308—C307—P3	119.3 (3)
P1—C2—H2	121.5 (19)	C309—C308—C307	120.3 (4)
P3—C3—P4	97.54 (12)	C309—C308—H308	119.9
P3—C3—H3A	112.3	C307—C308—H308	119.9
P4—C3—H3A	112.3	C310—C309—C308	120.5 (4)
P3—C3—H3B	112.3	C310—C309—H309	119.8
P4—C3—H3B	112.3	C308—C309—H309	119.8
H3A—C3—H3B	109.9	C309—C310—C311	120.9 (4)
C5—C4—C9	118.4 (2)	C309—C310—H310	119.6
C5—C4—N1	120.9 (2)	C311—C310—H310	119.6
C9—C4—N1	120.7 (2)	C310—C311—C312	119.2 (4)
C6—C5—C4	121.0 (3)	C310—C311—H311	120.4
C6—C5—H5	119.5	C312—C311—H311	120.4
C4—C5—H5	119.5	C307—C312—C311	120.6 (4)
C5—C6—C7	119.7 (3)	C307—C312—H312	119.7
C5—C6—H6	120.1	C311—C312—H312	119.7
C7—C6—H6	120.1	C402—C401—C406	119.1 (2)
C8—C7—C6	120.9 (3)	C402—C401—P4	119.99 (19)
C8—C7—Cl1	120.0 (2)	C406—C401—P4	120.9 (2)
C6—C7—Cl1	119.1 (3)	C403—C402—C401	120.8 (3)
C7—C8—C9	119.7 (3)	C403—C402—H402	119.6
C7—C8—H8	120.1	C401—C402—H402	119.6
C9—C8—H8	120.1	C402—C403—C404	119.7 (3)
C8—C9—C4	120.2 (3)	C402—C403—H403	120.1
C8—C9—H9	119.9	C404—C403—H403	120.1
C4—C9—H9	119.9	C405—C404—C403	120.0 (3)
C106—C101—C102	118.1 (2)	C405—C404—H404	120.0
C106—C101—P1	124.0 (2)	C403—C404—H404	120.0
C102—C101—P1	117.6 (2)	C404—C405—C406	120.6 (3)
C103—C102—C101	120.6 (3)	C404—C405—H405	119.7
C103—C102—H102	119.7	C406—C405—H405	119.7
C101—C102—H102	119.7	C405—C406—C401	119.8 (3)
C104—C103—C102	120.3 (3)	C405—C406—H406	120.1
C104—C103—H103	119.8	C401—C406—H406	120.1
C102—C103—H103	119.8	C408—C407—C412	119.1 (3)
C105—C104—C103	119.6 (3)	C408—C407—P4	119.3 (2)
C105—C104—H104	120.2	C412—C407—P4	121.1 (2)
C103—C104—H104	120.2	C407—C408—C409	120.1 (3)
C104—C105—C106	120.6 (3)	C407—C408—H408	119.9
C104—C105—H105	119.7	C409—C408—H408	119.9
C106—C105—H105	119.7	C410—C409—C408	119.5 (3)
C105—C106—C101	120.7 (3)	C410—C409—H409	120.3
C105—C106—H106	119.6	C408—C409—H409	120.3
C101—C106—H106	119.6	C411—C410—C409	120.8 (3)
C108—C107—C112	118.6 (2)	C411—C410—H410	119.6
C108—C107—P1	120.4 (2)	C409—C410—H410	119.6
C112—C107—P1	120.99 (19)	C410—C411—C412	119.7 (3)
C109—C108—C107	120.7 (3)	C410—C411—H411	120.2
C109—C108—H108	119.6	C412—C411—H411	120.2
C107—C108—H108	119.6	C411—C412—C407	120.8 (3)
C110—C109—C108	120.0 (3)	C411—C412—H412	119.6
C110—C109—H109	120.0	C407—C412—H412	119.6
C108—C109—H109	120.0	N4—C10—Ir1	178.1 (2)
C109—C110—C111	119.8 (3)	C12—O2—H2A	109.5
C109—C110—H110	120.1	O2—C12—H12A	109.5
C111—C110—H110	120.1	O2—C12—H12B	109.5
C110—C111—C112	120.5 (3)	H12A—C12—H12B	109.5
C110—C111—H111	119.8	O2—C12—H12C	109.5
C112—C111—H111	119.8	H12A—C12—H12C	109.5
C111—C112—C107	120.4 (3)	H12B—C12—H12C	109.5
C111—C112—H112	119.8	C11—O1—H1A	109.5
C107—C112—H112	119.8	O1—C11—H11A	109.5
C206—C201—C202	119.1 (3)	O1—C11—H11B	109.5
C206—C201—P2	121.1 (2)	H11A—C11—H11B	109.5
C202—C201—P2	119.7 (2)	O1—C11—H11C	109.5
C203—C202—C201	120.0 (3)	H11A—C11—H11C	109.5
C203—C202—H202	120.0	H11B—C11—H11C	109.5
			
C4—N1—N2—N3	−169.9 (2)	C203—C204—C205—C206	−0.5 (5)
Ir1—N1—N2—N3	−14.0 (3)	C204—C205—C206—C201	1.6 (4)
N1—N2—N3—C1	−1.1 (3)	C202—C201—C206—C205	−1.3 (4)
N2—N3—C1—P2	−111.2 (2)	P2—C201—C206—C205	−178.3 (2)
N2—N3—C1—Ir1	14.9 (3)	C2—P2—C207—C208	−38.4 (3)
C2—P2—C1—N3	118.60 (17)	C201—P2—C207—C208	81.5 (2)
C201—P2—C1—N3	−5.6 (2)	C1—P2—C207—C208	−161.0 (2)
C207—P2—C1—N3	−114.89 (17)	C2—P2—C207—C212	144.0 (2)
C2—P2—C1—Ir1	−3.16 (17)	C201—P2—C207—C212	−96.0 (2)
C201—P2—C1—Ir1	−127.35 (14)	C1—P2—C207—C212	21.4 (3)
C207—P2—C1—Ir1	123.34 (13)	C212—C207—C208—C209	−0.4 (4)
C201—P2—C2—P1	120.99 (16)	P2—C207—C208—C209	−178.0 (2)
C207—P2—C2—P1	−126.49 (16)	C207—C208—C209—C210	−0.3 (5)
C1—P2—C2—P1	−3.0 (2)	C208—C209—C210—C211	0.1 (5)
C107—P1—C2—P2	131.02 (16)	C209—C210—C211—C212	0.8 (5)
C101—P1—C2—P2	−121.34 (17)	C210—C211—C212—C207	−1.5 (5)
Ir1—P1—C2—P2	7.02 (18)	C208—C207—C212—C211	1.2 (4)
C307—P3—C3—P4	108.45 (14)	P2—C207—C212—C211	178.8 (2)
C301—P3—C3—P4	−140.67 (13)	C307—P3—C301—C302	−129.3 (2)
Ir1—P3—C3—P4	−11.87 (12)	C3—P3—C301—C302	117.2 (2)
C407—P4—C3—P3	−112.44 (13)	Ir1—P3—C301—C302	10.6 (3)
C401—P4—C3—P3	140.52 (13)	C307—P3—C301—C306	51.9 (3)
Ir1—P4—C3—P3	12.02 (12)	C3—P3—C301—C306	−61.6 (3)
N2—N1—C4—C5	−151.0 (2)	Ir1—P3—C301—C306	−168.3 (2)
Ir1—N1—C4—C5	56.8 (3)	C306—C301—C302—C303	−2.4 (5)
N2—N1—C4—C9	31.9 (3)	P3—C301—C302—C303	178.8 (3)
Ir1—N1—C4—C9	−120.4 (2)	C301—C302—C303—C304	1.5 (5)
C9—C4—C5—C6	0.3 (4)	C302—C303—C304—C305	0.8 (6)
N1—C4—C5—C6	−176.9 (2)	C303—C304—C305—C306	−2.1 (6)
C4—C5—C6—C7	0.0 (4)	C304—C305—C306—C301	1.2 (6)
C5—C6—C7—C8	−0.6 (4)	C302—C301—C306—C305	1.0 (5)
C5—C6—C7—Cl1	179.9 (2)	P3—C301—C306—C305	179.9 (3)
C6—C7—C8—C9	0.8 (5)	C301—P3—C307—C312	−137.7 (3)
Cl1—C7—C8—C9	−179.6 (2)	C3—P3—C307—C312	−25.8 (3)
C7—C8—C9—C4	−0.5 (4)	Ir1—P3—C307—C312	78.4 (3)
C5—C4—C9—C8	0.0 (4)	C301—P3—C307—C308	49.5 (3)
N1—C4—C9—C8	177.2 (3)	C3—P3—C307—C308	161.3 (2)
C2—P1—C101—C106	−112.8 (2)	Ir1—P3—C307—C308	−94.4 (2)
C107—P1—C101—C106	0.8 (3)	C312—C307—C308—C309	−1.1 (5)
Ir1—P1—C101—C106	125.1 (2)	P3—C307—C308—C309	171.9 (3)
C2—P1—C101—C102	60.8 (2)	C307—C308—C309—C310	0.8 (6)
C107—P1—C101—C102	174.4 (2)	C308—C309—C310—C311	0.1 (6)
Ir1—P1—C101—C102	−61.4 (2)	C309—C310—C311—C312	−0.7 (6)
C106—C101—C102—C103	0.6 (4)	C308—C307—C312—C311	0.5 (5)
P1—C101—C102—C103	−173.4 (2)	P3—C307—C312—C311	−172.4 (3)
C101—C102—C103—C104	−0.2 (4)	C310—C311—C312—C307	0.4 (6)
C102—C103—C104—C105	−0.5 (5)	C407—P4—C401—C402	−159.6 (2)
C103—C104—C105—C106	0.8 (5)	C3—P4—C401—C402	−49.0 (3)
C104—C105—C106—C101	−0.4 (5)	Ir1—P4—C401—C402	56.4 (3)
C102—C101—C106—C105	−0.3 (4)	C407—P4—C401—C406	16.7 (3)
P1—C101—C106—C105	173.3 (3)	C3—P4—C401—C406	127.4 (2)
C2—P1—C107—C108	0.8 (3)	Ir1—P4—C401—C406	−127.2 (2)
C101—P1—C107—C108	−109.9 (2)	C406—C401—C402—C403	−1.1 (4)
Ir1—P1—C107—C108	122.2 (2)	P4—C401—C402—C403	175.3 (2)
C2—P1—C107—C112	179.9 (2)	C401—C402—C403—C404	0.7 (5)
C101—P1—C107—C112	69.2 (2)	C402—C403—C404—C405	0.5 (5)
Ir1—P1—C107—C112	−58.7 (2)	C403—C404—C405—C406	−1.2 (5)
C112—C107—C108—C109	0.4 (4)	C404—C405—C406—C401	0.8 (5)
P1—C107—C108—C109	179.4 (2)	C402—C401—C406—C405	0.3 (4)
C107—C108—C109—C110	−0.8 (5)	P4—C401—C406—C405	−176.1 (2)
C108—C109—C110—C111	0.3 (5)	C401—P4—C407—C408	−93.6 (2)
C109—C110—C111—C112	0.6 (5)	C3—P4—C407—C408	158.6 (2)
C110—C111—C112—C107	−1.0 (5)	Ir1—P4—C407—C408	52.6 (3)
C108—C107—C112—C111	0.5 (4)	C401—P4—C407—C412	78.8 (3)
P1—C107—C112—C111	−178.6 (2)	C3—P4—C407—C412	−29.0 (3)
C2—P2—C201—C206	167.1 (2)	Ir1—P4—C407—C412	−135.1 (2)
C207—P2—C201—C206	45.1 (2)	C412—C407—C408—C409	0.6 (4)
C1—P2—C201—C206	−71.0 (2)	P4—C407—C408—C409	173.1 (2)
C2—P2—C201—C202	−9.8 (2)	C407—C408—C409—C410	1.9 (5)
C207—P2—C201—C202	−131.8 (2)	C408—C409—C410—C411	−2.6 (5)
C1—P2—C201—C202	112.1 (2)	C409—C410—C411—C412	0.8 (5)
C206—C201—C202—C203	−0.1 (4)	C410—C411—C412—C407	1.7 (5)
P2—C201—C202—C203	176.9 (2)	C408—C407—C412—C411	−2.4 (5)
C201—C202—C203—C204	1.2 (4)	P4—C407—C412—C411	−174.8 (3)
C202—C203—C204—C205	−0.9 (5)		

### {4-[3-(4-Chlorophenyl)triazenido-κN3]-1,1,3,3-tetraphenyl-1,3λ5-diphospha-κP1-but-2-en-4-yl}cyanido[methylenebis(diphenylphosphine)-κ2P,P']iridium(III) methanol disolvate (3) Hydrogen-bond geometry (Å, º)

**Table:**

*D*—H···*A*	*D*—H	H···*A*	*D*···*A*	*D*—H···*A*
O1—H1*A*···N4	0.83	2.02	2.8181 (1)	162
O2—H2*A*···N3	0.83	2.16	2.9486 (1)	158
